# Early Oral Administration of D-Chiro-Inositol Reverses Hippocampal Insulin and Glutamate Signaling Deficits in the 3×Tg Humanized Mouse Model of Alzheimer’s Disease

**DOI:** 10.3390/nu17183024

**Published:** 2025-09-22

**Authors:** Beatriz Pacheco-Sánchez, Julia Verheul-Campos, Antonio Vargas, Rubén Tovar, Miguel Rodríguez-Pozo, Juan A. Navarro, Antonio J. López-Gambero, Elena Baixeras, Pedro J. Serrano-Castro, Juan Suárez, Carlos Sanjuan, Patricia Rivera, Fernando Rodríguez de Fonseca

**Affiliations:** 1 Grupo de Neuropsicofarmacología, Instituto de Investigación Biomédica de Málaga y Plataforma en Nanomedicina-IBIMA Plataforma BIONAND, Unidad Clínica de Neurología, 29010 Málaga, Spain; beatriz.pacheco@ibima.eu (B.P.-S.); verheuljulia@gmail.com (J.V.-C.); antonio.vargas@ibima.eu (A.V.); rubentovar14@gmail.com (R.T.); rpmiguel@uma.es (M.R.-P.); juan_naga@hotmail.es (J.A.N.); antonio-jesus.lopez-gambero@inserm.fr (A.J.L.-G.); patricia.rivera@ibima.eu (P.J.S.-C.); juan.suarez@uma.es (J.S.); riveragonzalezpatricia@uma.es (P.R.); 2 Facultad de Ciencias, Universidad de Málaga, 29010 Málaga, Spain; 3 Departamento de Anatomía Humana, Medicina Legal e Historia de la Ciencia, Facultad de Medicina, Universidad de Málaga, 29071 Málaga, Spain; 4 Departamento de Bioquímica, Biología Molecular e Inmunología, Facultad de Medicina, Universidad de Málaga, 29010 Málaga, Spain; ebaixeras@uma.es; 5 Andalusian Network for Clinical and Translational Research in Neurology [NEURO-RECA], 29001 Málaga, Spain; 6 Euronutra S.L., Calle Johannes Kepler, 3, 29590 Málaga, Spain; euronutra@euronutra.eu

**Keywords:** Alzheimer’s disease, brain insulin resistance, D-chiro-inositol, glutamate receptors, hippocampus, proteomics, 3×Tg-AD mouse model

## Abstract

**Background and Objective:** Humanized models of Alzheimer’s disease (AD) provide valuable tools for investigating the mechanisms of this neurodegenerative disorder, the leading cause of dementia. These models enable the study of AD progression and the potential disease-modifying properties of drugs or dietary nutrients delivered through nutrition. Here, we examine molecular markers of metabolic and synaptic dysfunction in the hippocampus of 6-month-old 3×Tg-AD mice and assess whether a dietary insulin sensitizer can delay synaptic decline. **Methods**: First we characterized the molecular phenotype of 3×Tg-AD at 12 months using shotgun proteomics and phosphoproteomics to assess metabolic and synaptic changes in the hippocampus. Then, we characterized the effects of early daily oral D-chiro-inositol (DCI, *Gyneos*^®^) for three months, starting at 3 months of age, to test restoration of insulin signaling and glutamatergic synaptic markers. To this end we evaluated a) insulin signaling pathway components (insulin receptor, IRS1, PI3K, AKT, GSK3β) at mRNA, protein, and phosphorylation levels, and b) the expression of glutamate receptors (mGluR5, GluR1, GluR2, NMDAR1, NMDAR2A, NMDAR2B). Sex effects were explored. **Results**: 12-month 3×Tg-AD mice exhibit metabolic and synaptic dysfunction in the hippocampus, with phosphoproteomic changes suggesting altered glutamatergic synapses. At 6 months, disruptions in insulin signaling were evident, including altered expression and phosphorylation of insulin pathway components, and changes in glutamate receptor subunits. Early DCI treatment largely reversed these alterations. Several effects showed sex dependency. **Conclusions:** Early insulin-sensitizing intervention via DCI can restore insulin signaling and counteract hippocampal synaptic impairments in this AD model, supporting the potential for nutrient-based strategies to delay synaptic decline. Sex differences underscore the need to tailor therapeutic approaches in modifying AD progression.

## 1. Introduction

Alzheimer’s disease (AD) is a progressive neurodegenerative disorder and the leading cause of severe cognitive impairment and dementia worldwide. According to the World Health Organization (WHO), more than 55 million people are currently living with dementia, with AD accounting for 60–70% of cases [1]. While age is the strongest known risk factor for AD, other contributing factors include insulin resistance, neuroinflammation, dysregulation of glutamatergic and cholinergic neurotransmission, associated with the accumulation of pathological protein aggregates such as amyloid beta (Aβ) plaques and hyperphosphorylated tau neurofibrillary tangles (NFTs) in brain tissues [2,3,4,5,6]. Despite recent advances, including FDA-approved immunotherapies targeting Aβ, no definitive cure exists, and available treatments provide only symptomatic relief [7]. Given the multifactorial nature of AD, research is increasingly focused on interventions targeting metabolic dysfunction and neurotransmitter imbalances to mitigate cognitive decline [7,8].

One emerging therapeutic avenue involves the modulation of insulin signaling, as insulin resistance is increasingly recognized as a central contributor to AD pathophysiology. Often referred to as “type 3 diabetes” [9], AD has been linked to impaired insulin signaling, which disrupts glucose metabolism, neuronal survival, and synaptic plasticity, thereby exacerbating neurodegeneration [6,9,10,11]. Insulin signaling in the central nervous system (CNS) is mediated through the phosphoinositide 3-kinase (PI3K)/AKT pathway, which is critical for energy homeostasis, neurotransmission, and synaptic integrity [12,13]. Defects in this pathway have been observed in AD patients and are associated with neuronal energy deficits, oxidative stress, and increased vulnerability to Aβ toxicity [12,13,14]. Furthermore, insulin resistance impacts glutamatergic neurotransmission, particularly through dysregulation of N-methyl-D-aspartate receptors (NMDARs), contributing to excitotoxicity, synaptic dysfunction and cognitive impairment [15,16,17].

In this context, insulin sensitizers such as D-chiro-inositol (DCI) represent a promising intervention for restoring insulin signaling in AD. DCI is a naturally occurring inositol isomer that can be incorporated into the body through diet and plays a crucial role in insulin-mediated glucose uptake and metabolism by acting through the PI3K/AKT pathway [18,19]. In addition to improving insulin sensitivity, DCI has been implicated in modulating neuroinflammatory responses and oxidative stress, which are key drivers of AD pathology [20,21,22]. Given its role in metabolic regulation, PI3K/AKT signaling [23], DCI may also influence glutamatergic signaling by stabilizing NMDAR function, potentially reducing excitotoxicity and synaptic deficits observed in AD. Preliminary observations indicate that DCI has beneficial effects in AD by counteracting the toxic actions of Abeta oligomers and by normalizing metabolic dysfunctions associated with amyloidosis in the humanized 5XFAD model of AD [24,25].

To evaluate the therapeutic potential of DCI in AD, we utilized the triple-transgenic (3×Tg-AD) mouse model, which harbors three human familial AD mutations (APP Swedish, MAPT P301L, and PSEN1 M146V) and exhibits progressive Aβ and tau pathology. This model recapitulates key aspects of AD, including Aβ deposition, tangles of hyperphosphorylated tau, synaptic dysfunction, and cognitive deficits [26]. Previous studies have identified alterations in the PI3K/AKT pathway in 3×Tg-AD mice, along with reduced insulin receptor sensitivity and increased susceptibility to neurodegeneration [27]. Additionally, these mice display aberrant glutamatergic transmission, with enhanced NMDAR activation leading to excitotoxicity, further contributing to cognitive decline [28].

In the present study, we aimed to assess the efficacy of DCI as a neuroprotective agent in the 3×Tg-AD mouse model by examining its effects on insulin signaling and glutamatergic receptor function. Specifically, we investigated whether DCI administration could reverse insulin resistance and restore PI3K/AKT signaling while modulating NMDAR activity to prevent excitotoxic damage. By targeting these interconnected pathological pathways, we hypothesize that DCI may offer a novel approach to mitigating cognitive decline in AD. Our findings provide insight into the therapeutic potential of inositol-based interventions in neurodegenerative diseases and highlight the need for further research into metabolic therapies for AD.

## 2. Materials and Methods

### 2.1. Animals and Ethics Statement

All experiments were realized in compliance with the ARRIVE guidelines [29] and in concordance with the European Communities Council Directives 2010/63/EU, Regulation (EC) no. 86/609/ECC (24 November 1986), and Spanish National and Regional Guidelines for Animal Experimentation (Real Decreto 53/2013). Experimental protocols were approved by The Local Ethical Committee for Animal Research of the University of Malaga (CEUMA no. 203-2023-A, 1 April 2024). Animals used in this experiment were non-transgenic (Non-Tg) and homozygous (3×Tg-AD) male and female mice. The 3×Tg-AD mouse model (Jackson Laboratory, Bar Harbor, ME, USA), which combines mutant hAPP (Swedish), PSEN1 (MM146V) and tau (P301L) transgenes, and results in Aβ and tau pathologies, was used in the present study [26]. The rodents were housed individually in the Animal Centre for Experimentation at the University of Malaga with water and food provided ad libitum under standardized conditions: a 12-h light/dark cycle, 20 ± 2 °C of room temperature, and 40 ± 5% of relative humidity. A first batch of animals of 10–12 months of age (5 female and 5 male wild-type; 5 female and 4 male 3×Tg-AD was used for proteomic studies. A second batch of animals of 6 months of age (12 WT and 12 3×Tg-AD, half males and half females) was used for plasma monitoring of DCI concentrations after acute oral administration. A third batch of animals, 12 female and 12 male wild-type; 12 female and 12 male 3×Tg-AD, was used for analyzing the effects of the oral administration of DCI for three consecutive months.

Accordingly, all efforts were made to minimize animal suffering and to reduce the number of animals used. Potential confounders, such as the order of treatments and measurements, and animal/cage location, were minimized by standardizing the timing of treatments and measurements for all animals and ensuring consistent environmental conditions across cages. Additionally, cage locations were rotated periodically to prevent location bias. These measures were implemented to reduce the influence of external variables on the study outcomes.

### 2.2. Pharmacological Treatment with Pure DCI and Monitorization of Its Plasma Levels

#### 2.2.1. Pharmacological Treatment with Pure DCI

For studies of DCI absorption through the oral route, 12 WT and 12 3×Tg-AD mice of 6 months of age (half males and half females), received an intragastric administration of DCI (200 mg/kg, *Gyneos^®^*, Euronutra S.L., Málaga, Spain), dissolved in a sterile water at 25 mg/mL, at a volume of 8 mL/kg. Animals were sacrificed at 0, 1 and 4 h after oral administration, and plasma was obtained and stored for monitoring DCI concentrations.

For studies addressing the brain effects of chronic oral administration of DCI, mice were fed with a standard pellet diet (3.02 Kcal/g with 30 Kcal% protein, 55 Kcal% carbohydrates, and 15 Kcal% fat; Harlam; Tecklad, Madison WI, USA) supplemented with DCI (*Gyneos*^®^-DCI, 98% purity; Cambridge MA, USA) at libitum in their drinking water for 12 weeks (3 months). All animals were about 12 weeks of age (3 months old) at the beginning of the experiment and 6 months old at the termination. Daily doses were 200 mg/kg/day, as a dissolved powder in water bottles (1 mg/mL, consuming 5 mL/day per 25 g of body weight). We established the following experimental groups: non-transgenic (WT-DCI (*Gyneos^®^*) n = 12; 6 males; 6 females) and transgenic 3×Tg-AD (n = 12; 6 males; 6 females) mice that received DCI (*Gyneos^®^*) as treatment. Control groups of both genotypes, non-transgenic wild-type (WT-CTR; n = 12; 6 males, 6 females) and 3×Tg-AD transgenic mice (3×Tg-CTR; n = 12; 6 males, 6 females), received water as the vehicle solution. The sample size was determined based on a balanced 2 × 2 experiment (Genotype: Control vs. KO; treatment: Vehicle vs. DCI) to detect the Genotype × Treatment interaction—i.e., whether treatment with DCI acts differently across genotypes. We assumed the KO baseline differs from Control by 35% and that DCI treatment normalizes KO to Control levels, with a common within-cell variability equal to 20% of the Control mean; both the effect size and variance were estimated from previous similar studies [30]. Using a two-sided α = 0.05 and 80% power, and analyzing the interaction via two-way ANOVA (difference-in-differences contrast), the required sample size is 11 animals per cell. To accommodate potential procedural losses, we added one animal per cell, yielding 12 animals per cell and a total of 48 animals across the four cells. All calculations were performed in GPower (version 3.1.9).

Finalized the DCI administration for 12 weeks, the animals were anaesthetized at 24 weeks of age (6 months old) with intraperitoneal sodium pentobarbital injection (50 mg Kg^−1^ BW) 5 min before the mice were sacrificed. Tissue samples were rapidly removed and immediately frozen at −80 °C for later analysis (refer to Section 2.4), taking the whole process less than 10 min from the administration of pentobarbital.

#### 2.2.2. Measurement of Plasma Levels of DCI

Plasma DCI concentrations were determined by gas chromatography–mass spectrometry (GC/MS) employing D-Pinitol as the internal standard. All procedures were performed using analytical-grade reagents under controlled laboratory conditions. Plasma samples were thawed once, gently mixed, and aliquoted (21 μL). Metabolite extraction was achieved by adding 0.7 mL of a pre-cooled single-phase solvent mixture of isopropanol: acetonitrile: water (3:3:2, *v*/*v*) at 20 °C, followed by vortexing for 5 min. After centrifugation (10,000× *g*, 5 min, 4 °C), 0.5 mL of the clear supernatant was transferred into clean tubes and evaporated to dryness using a SpeedVac concentrator.

Derivatization Procedure: Dried extracts were derivatized in two sequential steps:

(a) Methoximation—Carbonyl groups were protected by adding 10 μL of methoxyamine hydrochloride solution (40 mg/mL in pyridine) and incubating at 30 °C for 90 min.
(b) Silylation—Volatility was enhanced by adding 80 μL of N-methyl-N-trimethylsilyltrifluoroacetamide (MSTFA) containing 1% trimethylchlorosilane (TMCS, Pierce) and incubating at 37 °C for 30 min.

Analyses were performed on an Agilent 8890 GC equipped with a mass selective detector (MSD). The oven was programmed from 60 °C (1 min hold) to 325 °C at 10 °C/min, with a final hold of 10 min, resulting in a total run time of 37.5 min including post-run cooling. A 1 μL sample volume was injected using a 10 μL syringe into a split/splitless injector set at 250 °C, under split mode (1:10 ratio). The carrier gas was helium at a constant flow rate of 1 mL/min. The MSD transfer line was maintained at 290 °C, the ion source at 230 °C, and the quadrupole at 150 °C. The instrument was operated in electron impact (EI) mode with a solvent delay of 3 min, scanning from *m*/*z* 50–550 at a data rate of 20 Hz. Quantification was performed in selected ion monitoring (SIM) mode: (a) DCI: target ion *m*/*z* 305; confirmatory ions *m*/*z* 265, 217, 191; (b) D-Pinitol: target ion *m*/*z* 305; confirmatory ions *m*/*z* 260, 217, 191.

Independent stock solutions of DCI and D-Pinitol (1000 μg/mL) were prepared in ultrapure water. Calibration standards were prepared by serial dilution in either ultrapure water or DCI-free blank plasma to final concentrations ranging from 76.92 to 0.105 μg/mL. All calibrators, quality control (QC) samples, and test samples were processed identically. The calibration curve was generated by plotting the peak area ratio (DCI/D-Pinitol) against nominal DCI concentrations. Linearity was evaluated in both aqueous and plasma matrices. Blank samples (water and plasma) were analyzed to assess endogenous interference. Data collection and processing were performed using MassHunter Workstation Software, version 10.1 (Agilent Technologies, St. Clara, CA, USA). Quantitative results were derived from linear regression of the calibration curves, applying appropriate weighting factors when necessary.

### 2.3. Tissue Sampling and Biochemical Procedures

#### 2.3.1. Sample Collection

Before sacrifice, animals were anesthetized, and blood was drawn directly from the right atrium. It was centrifuged (2100× *g* for 10 min) for plasma collection and it was kept at −80 °C for biochemical analysis of DCI. After that, animals were transcranial perfused with 0.1 M phosphate-buffered saline (PBS). Brain samples were rapidly removed and bisected down the midline and the hemispheres flash frozen in dry ice and stored at −80 °C for biochemical analysis. The right hemisphere was used for proteomics, and the left hemi-brain was used for real time PCR and Western blot analysis.

#### 2.3.2. Proteomic Analysis of Dorsal Hippocampus

##### Sample Preparation

Mice hippocampus tissues were homogenized and lysed in RIPA buffer (Sigma-Aldrich, St. Louis, MO, USA) containing phosphatase inhibitor (Halt™ Phosphatase Inhibitor Cocktail, Thermo Fisher Scientific) and nuclease (Pierce™ Universal Nuclease for Cell Lysis, Pierce, IL, USA). Proteins from the samples were purified, and gel-assisted proteolysis was carried out. Briefly, the protein solution was entrapped in a polyacrylamide gel matrix before reduction with dithiothreitol and cysteine residue carbamidomethylation with iodoacetamide. Then, the proteins were digested by trypsin (Promega, Madison, WI, USA), and peptides were extracted from the gel. For phosphoproteomic analysis, 45 micrograms of each hippocampal tryptic digest were subjected to Thermo Scientific™ Pierce™ HiSelect TiO_2_ Phosphopeptide Enrichment Kit (Thermo Fisher Scientific, Waltham, MA, USA) and the TiO_2_ eluent was saved for MS analysis.

##### LC-MS and Data Analysis

For the LC-MS analysis, samples were injected onto an Easy nLC 1200 UHPLC system coupled to a Q Exactive HF-X Hybrid Quadrupole-Orbitrap mass spectrometer (Thermo Fisher Scientific, Waltham, MA, USA). Data were acquired using Tune 2.9 and Xcalibur 4.1.31.9 (Thermo Fisher Scientific, Waltham, MA, USA). Peptides from the samples were automatically loaded into a trap column (Acclaim PepMap 100, 75 µm × 2 cm, C18, 3 µm, 100 A, Thermo Fisher Scientific) and eluted onto a 25 cm analytical column (PepMap RSLC C18, 2 µm, 100 A, 75 µm × 25 cm, Thermo Fisher Scientific). For phosphopeptide-enriched samples, the use of trap column was avoided. The binary gradient mobile phase consisted of 0.1% formic acid in water (solvent A) and 0.1% formic acid in 80% acetonitrile (solvent B). Peptides were eluted from the analytical column with a 180 min gradient from 2 to 20% solvent B, followed by a 30 min gradient from 20 to 35% solvent B and finally in 95% solvent B for 15 min before re-equilibration in 2% solvent B at a constant flow rate of 300 nL/min.

Data acquisition was performed in electrospray ionization positive mode. MS1 scans were acquired from *m*/*z* 375–1600 at a resolution of 120,000. Using a data-dependent acquisition method, the 15 most intense precursor ions with +2 to +6 charges were isolated within a 1.2 *m*/*z* window and fragmented to obtain the corresponding MS2 spectra. The fragment ions were generated in a higher-energy collisional dissociation cell with a fixed first mass at 110 *m*/*z* and detected by the Orbitrap mass analyzer at a resolution of 30,000.

The raw data were analyzed using Proteome Discoverer^TM^ 2.5 (Thermo Fisher Scientific, Waltham, MA, USA). Data were searched against *Mus musculus* UniProt database (2023-03-01 version) using the Sequest HT search engine with a precursor mass tolerance of 10 ppm and fragment mass tolerance of 0.02 Da. Carbamidomethylation (+57.021 Da) for cysteine was used as a fixed modification, and methionine oxidation (+15.996 Da) and phosphorylation (+79.966 Da, T, Y, S) as variable modifications. Protein assignments were validated using the Percolator algorithm [31] by imposing a strict cut-off of a 1% false discovery rate (FDR). Protein abundance ratios were calculated based on precursor intensities [31].

##### Data Processing: Selection of Normalization Methods

Raw data were first organized and pre-processed for intuitive filtering and then the outlier detection was conducted using SEAOP (Statistical Expression Analysis of Omics Profiles), where the k-nearest neighbors (KNN) algorithm identified and removed anomalous values that deviated significantly from group trends. This step minimized potential biases introduced by outliers, ensuring that subsequent analyses reflected biologically relevant expression patterns rather than technical variability. Normalization of the proteomic data was essential to adjust for sample-to-sample differences in total read counts, especially considering that some proteins showed high abundance, while others had extremely low expression levels. Various normalization methods were assessed, including (1) mean-based normalization, which adjusted each sample based on the average protein abundance, (2) down-scaling normalization factor, using the sample with the lowest total read count as a reference to prevent overestimations in high-abundance proteins, (3) total read count normalization, scaling each sample according to its sum of protein abundances, and (4) housekeeping protein normalization, using beta-actin and beta-tubulin. The down-scaling normalization was selected as it provided consistent results, with minimal distortion of relative expression levels, particularly preserving the biological signal of proteins expressed at lower levels. This method applied a correction factor calculated by dividing the lowest-read sample’s total by each sample’s total, applied independently for male and female groups to account for sex-specific variations.

##### Fold Change Calculation

To quantify changes in protein expression levels between treated and control groups, fold change values were calculated by dividing the mean abundances in each treated group by those in the control group. These ratios were log2-transformed, simplifying interpretation by converting large multiplicative differences into manageable additive values. Fold change values below −0.41 or above 0.32 were marked as significant changes, aligning with standard thresholds for differential expression analysis in proteomics. This approach allowed for a balanced view of both up- and downregulated proteins in response to alcohol and stress conditions. For proteomic analysis, statistical analyses were employed to identify significant differences in protein abundance between experimental groups. The normality of each dataset was tested using the Shapiro–Wilk test, which guided the choice of subsequent analyses. For normally distributed data, Student’s *t*-test was used to compare group means, whereas non-normally distributed data were analyzed with the Mann–Whitney U test to compare median values. Significance was set at *p* < 0.05, and proteins with a minimum change of 25% between groups were considered for further analysis. Additionally, *p*-values were adjusted to log10 for visualization purposes, while fold change values were log2-transformed to facilitate interpretation and enhance the comparability of up- and downregulated proteins across groups. These adjustments ensured that only the most statistically robust differences were retained, enhancing the biological relevance of the findings. In our initial approach, we aim to obtain the most comprehensive functional networks possible. Therefore, no adjustments will be applied at this stage to ensure that the interaction subgraphs remain as interconnected as possible. Adjustments will be applied during the candidate selection process to refine the analysis.

##### Results Visualization

To communicate the results effectively, several visualization techniques were utilized. Volcano plots were generated to display differentially expressed proteins, with selection criteria based on a 35% change rate, corresponding to log fold change values below −0.41 or above 0.32. Proteins meeting these thresholds were classified as significantly up- or downregulated. Parameters for differential expression thresholds were calculated using values from the bivariate analysis. To obtain interaction data, an API query to STRING was performed.

##### Complexity Reduction and Candidate Selection

To reduce complexity while preserving biological relevance, proteins were selected based on their relative variance, retaining those with high intergroup and low intragroup variance. This filtering emphasized candidates with consistent differentiation between experimental groups, yielding a focused set of proteins for downstream analysis. Proteins in the top 5% of relative variance were retained to maintain an optimal balance between data depth and interpretability. Further, a False Discovery Rate (FDR) correction was applied to *p*-values, reducing the risk of false positives and ensuring statistical rigor.

##### Functional Analysis

Functional enrichment analysis was conducted using Gene Ontology (GO) and Kyoto Encyclopedia of Genes and Genomes (KEGG) terms to categorize differentially expressed proteins into relevant biological processes, cellular components, and molecular functions. From each category, the top 20 terms with the lowest False Discovery Rate (FDR) were selected to ensure statistical rigor and focus on the most significant enrichments. The STRING database was employed to further enhance the interpretation of these enriched terms by identifying protein–protein interactions and mapping them onto known signaling pathways. Enrichment data were visualized with ggplot2, which provided clear illustrations of the most relevant biological pathways impacted by alcohol and stress exposure. This analysis provided insights into the broader physiological and molecular effects induced by the experimental conditions.

#### 2.3.3. RT-qPCR

We assessed hippocampal mRNA levels for genes involved in: (a) insulin signaling, (b) glutamate receptors, (c) glial markers, and (d) endocannabinoid-related pathways. Real-time PCR was conducted as previously described [24]. Briefly, total RNA was extracted from brain sections with Trizol, purified with an RNAeasy MinElute cleanup kit including DNase I treatment, and quantified to confirm A260/280 of 1.8–2.0. cDNA was synthesized from 1 µg RNA using the Transcriptor Reverse Transcriptase kit, with primers/probes from TaqMan Gene Expression Assays. Primer details are in Supplementary Table S1. Real-time qPCR was performed on a CFX96 system using the FAM-labeled TaqMan format, followed by melting curve analysis to verify single-product amplification.

#### 2.3.4. Western Blot Analysis

We performed Western blotting as previously described [32]. Briefly, frozen hemi-hippocampi were homogenized in RIPA buffer, incubated on ice, centrifuged, and protein concentration measured by Bradford assay. Samples (10–15 µg) were mixed with a loading buffer, heated, and run on 4–12% Bis-Tris gels, then transferred to nitrocellulose membranes.

Membranes were blocked with 5% BSA-TBST and probed overnight with primary antibodies (listed in Supplementary Table S2). After washing, HRP-conjugated secondary antibodies were applied, and signals were detected with chemiluminescence using a Chemi-Doc system. For phospho-detection, membranes were stripped and reprobed with total antibodies. Band intensities were quantified with ImageJ software Version 1.53f51, normalized to γ-Adaptin, and reported as ratios of phospho-protein to total protein and total protein to γ-Adaptin.

### 2.4. Statistical Analyses

All data are shown as mean ± SEM. Statistical analyses for PCR, Western blot, and multiplex data were performed in GraphPad Prism 9. Normality was checked with Shapiro–Wilk, and homogeneity of variance with Levene’s test. Analyses included groups with n ≥ 5. ANOVA (one-way for DC I time course, two-way for genotype × treatment, or three-way for sex × genotype × treatment in Supplementary Figures S1–S10) followed by Tukey’s post hoc tests. Post hoc tests were only run if the ANOVA showed a significant effect (*p* < 0.05) and variances were homogeneous. Significance was set at *p* ≤ 0.05; non-significant results are *p* > 0.05.

To mitigate Type I error from multiple outcomes, we conducted power analyses and sample size estimations a priori. Effect sizes were inferred from prior literature to ensure adequate power, and adjustments for multiple comparisons were considered in the analyses.

## 3. Results

### 3.1. Proteomic Analysis of Dorsal Hippocampus in 3×Tg-AD Mice

**Figure 1:**
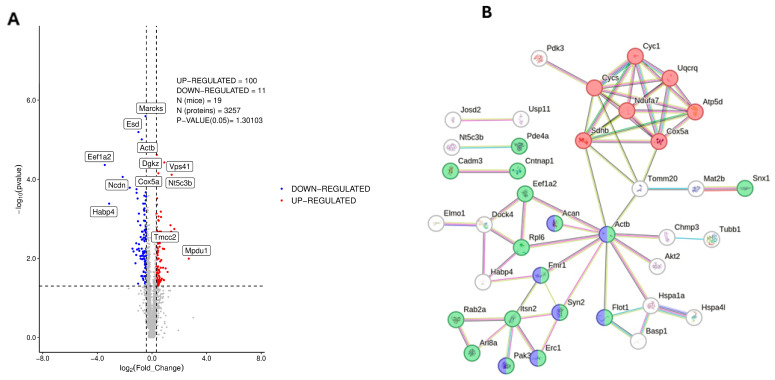
Deregulated expression of hippocampal proteins in the dorsal hippocampus of 3×Tg-AD mice, as measured by shotgun proteomics. Results were obtained from animals of 10–12 months of age (5 female and 5 male wild-type; 5 female and 4 male 3×Tg-AD). (**A**) Volcano plot showing up-regulated (right) and down-regulated (left) proteins in 3×Tg-AD with respect to wild-type animals. (**B**) STRING association network diagram of deregulated hippocampal proteins that are functionally associated. Red dots correspond to proteins related to oxidative phosphorylation. Green dots are proteins related to synapses, with the blue labelled present in glutamatergic synapses. Further details are provided in the main text.

In order to examine the previously described alterations in hippocampal protein expression in the 3×Tg-AD mice, we performed shotgun proteomics in 12-month-old male and female mice of both genotypes (Figure 1A,B, Supplementary Figure S1). Of the 3257 proteins detected, 100 proteins were found to be upregulated and 111 downregulated in 3×Tg-AD mice with respect to the wild-type genotype (Figure 1A). Gene ontology indicates that these proteins belong mostly to organelles, membranes and endomembranes (Supplementary Figure S1). A functional association analysis revealed a specific network of highly dysregulated proteins comprising mitochondrial oxidative phosphorylation (Cyc1, Cycs, Sdhb, Cox5a, Uqcrq, Ndufa7 and Atp5d). They are all core subunits of the mitochondrial respiratory chain and this pattern of dysregulation is a hallmark of mitochondrial dysfunction observed in aging, Alzheimer’s disease, and other neurodegenerative conditions—particularly in females, where estrogen decline reduces mitochondrial resilience.

Another functionally associated network comprises a dysregulated network of synaptic proteins specifically enriched in glutamatergic neurons—including Snx1, Flot1, Actb, Pde4a, Cadm3, Cntnap1, Eef1a2, Acan, Rpl6, Fmr1, Syn2, Rab2a, Itsn2, Arl8a, Pak3, and Erc1—points to a broad disturbance in synaptic structure, trafficking, and signaling. Many of these proteins are involved in vesicle recycling and trafficking (Snx1, Rab2a, Itsn2, Arl8a), actin cytoskeleton organization and dendritic spine stability (Actb, Pak3), presynaptic scaffolding and neurotransmitter release (Syn2, Erc1), as well as cell adhesion and axon guidance (Cadm3, Cntnap1). Others regulate intracellular signaling cascades that shape synaptic plasticity (Pde4a, Fmr1) or protein translation at the synapse (Eef1a2, Rpl6). Finally, the isoform AKT2 is markedly upregulated, indicating a disruption of the insulin signaling pathway in the hippocampus. (Figure 1B).

### 3.2. Phosphoproteomic Analysis of Dorsal Hippocampus in 3×Tg-AD Mice

**Figure 2:**
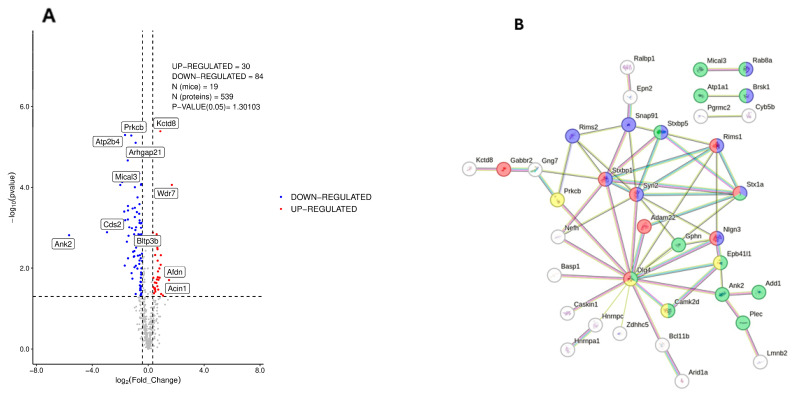
Deregulated expression of hippocampal phosphoproteins in the dorsal hippocampus of 3×Tg-AD mice, as measured by shotgun proteomics. Results were obtained from animals of 10–12 months of age (5 female and 5 male wild-type; 5 female and 4 male 3×Tg-AD). (**A**) Volcano plot showing up-regulated (right) and down-regulated (left) proteins in 3×Tg-AD with respect to wild-type animals. (**B**) STRING association network diagram of deregulated hippocampal phosphoproteins that are functionally associated. Red dots correspond to proteins present in glutamatergic synapses. Yellow dots are proteins related to the trafficking of AMPA glutamate receptors. Blue dots are proteins related to vesicle-mediated transport in synapses and green dots are cytoskeletal proteins. Further details are provided in the main text.

Phosphoproteome analysis also revealed a marked dysregulation of phosphorylated proteins in the hippocampus of 3×Tg-AD mice, with respect to wild-type genotype (Figure 2A,B, Supplementary Figure S2). Of the 539 proteins detected, 30 proteins were found to be upregulated and 84 downregulated in 3×Tg-AD mice with respect to the wild-type genotype (Figure 2A). Gene ontology indicates that these proteins belong mostly to organelles, membrane and endomembranes (Supplementary Figure S2). A functional association analysis using STRING (Figure 2A) revealed a specific network of proteins present again at glutamatergic synapses (Adam22, STX1a, Rims1, Nlgn3 and Gabbr2), including trafficking of AMPA glutamate receptors (Dlg4, Epb41l1, Camk4 or Prkcb). Other proteins are related to vesicle-mediated transport in synapses, including Rims2, Snap91, Brsk1 or Rab8A. Lastly, others are cytoskeletal proteins, such as Ank2, Plec, Gphn, Add1, Mical3 or Atp1a1. Finally, AKT2, a major metabolic sensor in the canonic insulin pathway, was found to be upregulated 2.2 times, suggesting a dysregulation of insulin signaling.

### 3.3. Plasma Concentrations of DCI After an Oral Administration

**Figure 3:**
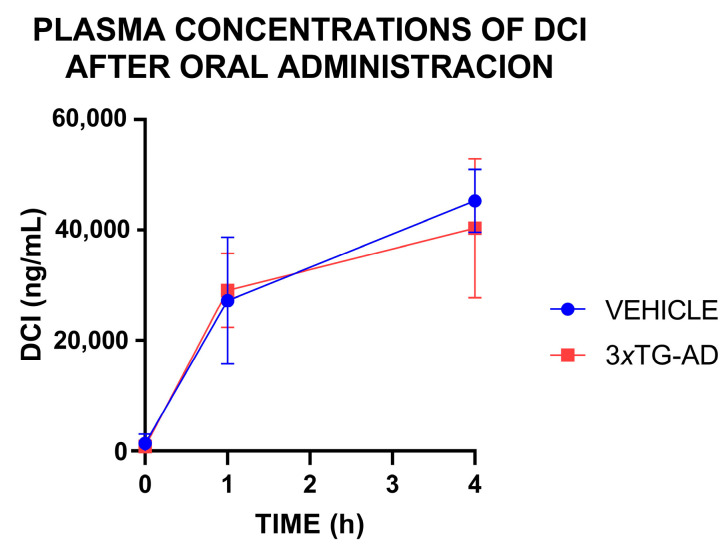
Plasma concentration of D-chiro-inositol (DCI) at 1 and 4 h after oral administration of a single dose of DCI (200 mg/kg) in wild-type and 3×Tg-AD mice. Data are means of 6 samples (3 males + 3 females) per genotype.

Plasma concentrations of DCI rose 1 h after its oral administration, with the levels sustained for at least 4 h (Time effect, *F*(1,22) = 86.84, *p* < 0.0001). Concentrations were found to be similar in both wild-type and 3×Tg-AD genotypes (genotype effect: *F*(1,22) = 0.19, *p* = 0.662, non-significant). These results indicate that DCI absorption and elimination allow stable levels, and that the administration of DCI in the drinking water will allow the sustained incorporation of this inositol into the blood stream. (See Figure 3).

### 3.4. Effects of DCI on the mRNA Expression of Insulin Receptor-PI3K/AKT Pathway in the Hippocampus of 3×Tg-AD Mice

**Figure 4:**
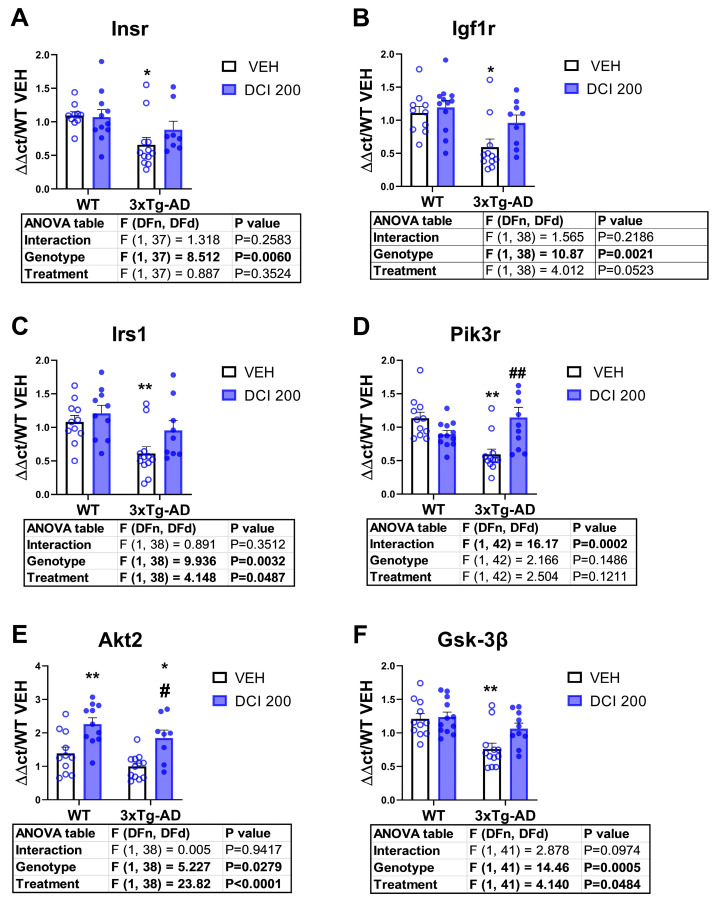
mRNA expression of the insulin receptor signaling pathway in the hippocampus of either, wild-type or 3×Tg-AD mice treated for 3 months with D-chiro-inositol (DCI). Real-time PCR measurement of mRNA expression of (**A**) the insulin receptor (Insr), (**B**) the insulin-like growth factor 1 receptor (Igf1r), (**C**) the insulin receptor substrate 1 (Irs1), (**D**) the phosphatidylinositol 3 kinase regulatory subunit (Pik3r), (**E**) AKT2 (Akt2) and (**F**) glycogen synthase kinase 3β (Gsk-3β) after 3 months of DCI treatment. Histograms represent mean ± SEM (n = 10–12 samples per group, half males and half females). Two-way ANOVA and Tukey’s test for multiple comparisons were performed: (*) *p* < 0.05, (**) *p* < 0.01, versus WT-VEH group; (#) *p* < 0.05, (##) *p* < 0.01 versus 3×Tg-VEH group.

The 3×Tg-AD mice exhibited a significant decrease in mRNA expression of either the Insulin receptor, the IGF-1 receptor and the insulin substrate receptor 1. Post hoc analysis revealed that these effects were reversed by DCI treatment (See Figure 4A–C respectively). In the case of PI3K mRNA expression, DCI produced a significant interaction, resulting in an upregulation of mRNA expression only in the 3×Tg-AD group (Figure 4D). Treatment with DCI resulted in a marked increase in the expression of AKT2 in both genotypes (Figure 4E). Finally, 3×Tg-AD mice also exhibited a decreased expression of the mRNA coding for GSK-3β, an effect also reversed by DCI (Figure 4F). Overall, 3×Tg-AD animals exhibited a downregulation of the mRNA expression of the canonical insulin signaling pathway in the dorsal hippocampus, an effect largely reversed by DCI treatment.

### 3.5. Effects of DCI on the Phosphorylation Status of the Main Proteins of the Canonical Insulin Signaling Pathway in the Hippocampus of 3×Tg-AD Mice

**Figure 5:**
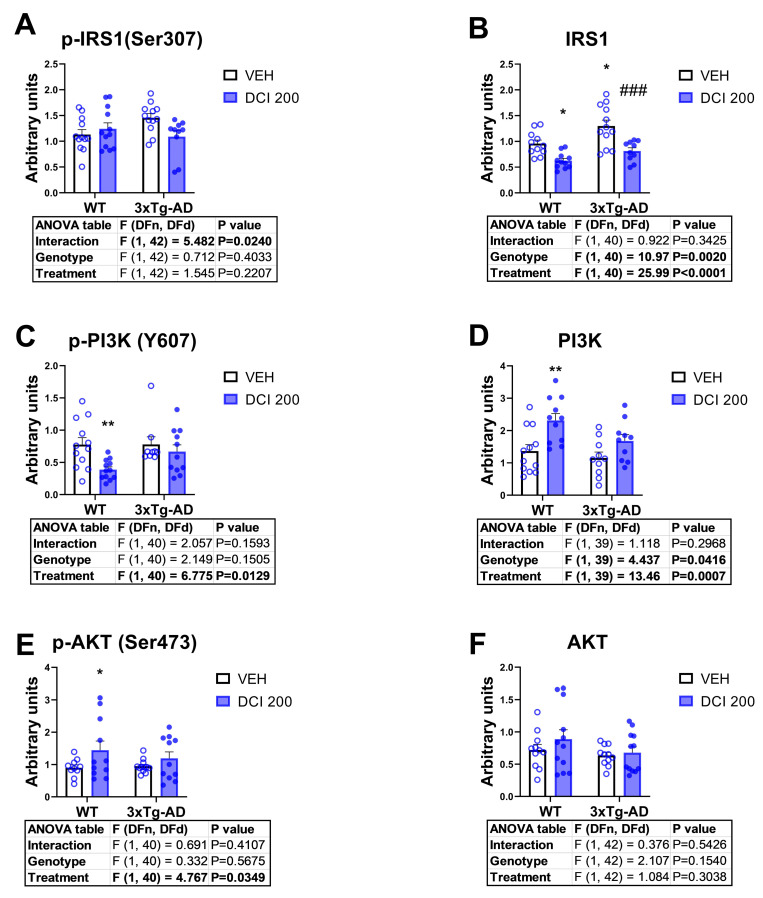

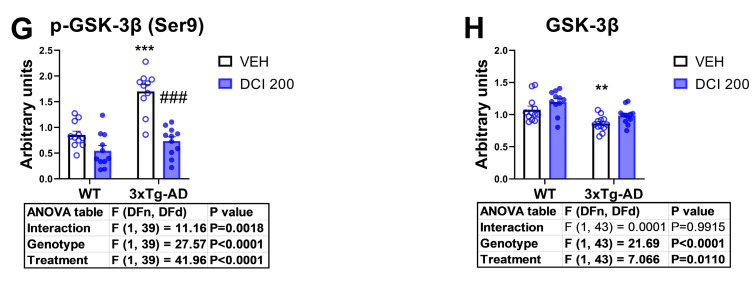
Functional status of the insulin receptor signaling pathway in the hippocampus of either, wild-type or 3×Tg-AD mice treated for 3 months with D-chiro-inositol (DCI). Western blot analysis of the phosphorylation status of (**A**) the insulin receptor substrate 1 (Irs1) protein, phosphorylated in the serine 307; (**B**) the total insulin receptor substrate 1 (Irs1) protein, (**C**) the phosphatidylinositol 3 kinase (p85-PI3K) phosphorylation at tyrosine 607, (**D**) the quantity of total p85-PI3K; (**E**) the protein Kinase B (AKT) phosphorylation on serine 473, (**F**) the amount of total AKT, (**G**) glycogen synthase kinase 3β (GSK-3β) phosphorylation at serine 9 and finally, (**H**) the amount of total GSK-3β. Representative blots can be found in Supplementary Figure S3. Samples were obtained simultaneously and processed in parallel. Histograms represent mean ± SEM (n = 10–12 animals per group, half males/half females). Two-way ANOVA and Tukey’s test for multiple comparisons were performed (*) *p* < 0.05, (**) *p* < 0.01, (***) *p* < 0.001, versus WT-VEH group; (###) *p* < 0.001 versus 3×Tg-AD-VEH group.

Two-way ANOVA indicated a genotype effect on protein expression of GSK-3β (ser 9), PI3K, and GSK3β; thus, Western blot analysis revealed that transgenic 3×Tg-AD mice only exhibited a marked phosphorylation of GSK-3β at serine 9, as a basal phenotypic finding (Figure 5A,C,E,G). However, they do exhibit greater basal contents of Irs1 (Figure 5B), and lower amounts of PI3K (Figure 5D) and GSK-3β (Figure 5H). In 3×Tg-AD mice, treatment with DCI tends to reduce the inhibitory phosphorylation of Irs-1 at Serine 307, without affecting PI3K/AKT kinases activation and reducing notably the increased phosphorylation of GSK-3β at serine 9 (Figure 5A,C,E,G). DCI (treatment effect in two-way ANOVA analysis) also reduced total Irs1, enhanced the total amount of PI3K, without modifying the total expression of GSK-3β (Figure 5B,D,F,H).

### 3.6. Effects of DCI on Glial Markers in the Hippocampus of 3×Tg-AD Mice

**Figure 6:**
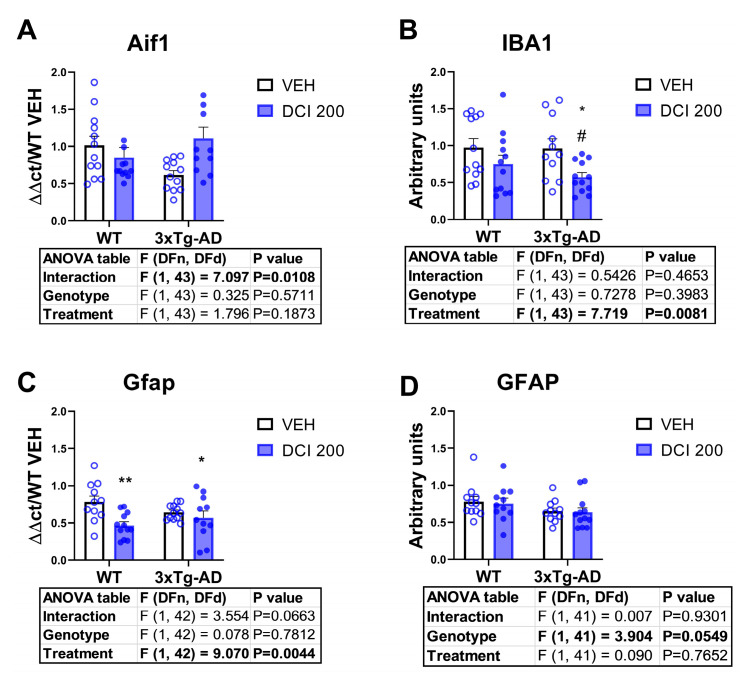
Effects of DCI treatment on the expression of glial markers in the hippocampus of either, wild-type or 3×Tg-AD mice. (**A**) mRNA expression of the microglial marker Aif1 and, (**B**) expression of Aif1-translated microglial protein Iba1. (**C**) mRNA expression of the astrocytic gene marker Gfap and (**D**) protein expression of Gfap as measured by Western blot. Corresponding blots can be found in the Supplementary Figure S4. Samples were obtained simultaneously and processed in parallel. Histograms represent mean ± SEM (n = 10–12 animals per group, half males/half females). Two-way ANOVA and Tukey’s test for multiple comparisons were performed. (*) *p* < 0.05, (**) *p* < 0.01 versus WT-VEH group; (#) *p* < 0.05, versus 3×Tg-AD-VEH group.

In order to evaluate the status of glial reactivity markers, we measured the mRNA expression and the protein expression of Aif1/Iba1 for microglia (Figure 6A,B), and Gfap for astrocytes (Figure 6C,D). Data indicate that there was no genotype effect in any of the markers (although there was an almost significant decrease of Gfap protein in 3×Tg-AD, Figure 6D). Treatment with DCI (treatment effect in two-way ANOVA analysis) resulted in a genotype-independent decrease in protein expression of Iba1 (Figure 6B) and a decrease in the mRNA expression of Gfap, which was centered on wild-type genotype (Almost significant treatment × genotype interaction, Figure 6C).

### 3.7. Effects of DCI on the mRNA Expression of Glutamate Receptors in the Hippocampus of 3×Tg-AD Mice

**Figure 7:**
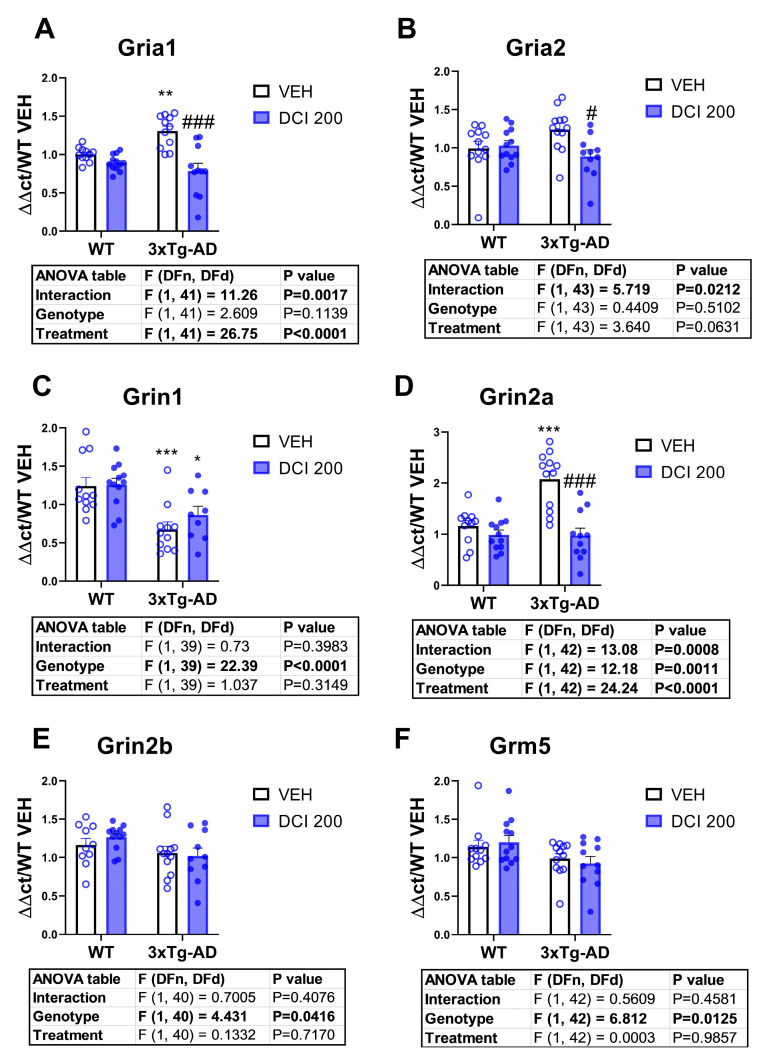
mRNA expression of glutamate receptors in the hippocampus of either wild-type or 3×Tg-AD mice treated for 3 months with D-chiro-inositol (DCI). Real-time PCR measurement of mRNA expression of (**A**) glutamate ionotropic Receptor AMPA Type Subunit 1 (Gria1), (**B**) Glutamate Ionotropic Receptor AMPA Type Subunit 2 (Gria2), (**C**) the glutamate ionotropic receptor NMDA type subunit 1 (Grin1), (**D**) the glutamate ionotropic receptor NMDA type subunit 2a (Grin2a), (**E**) the glutamate ionotropic receptor NMDA type subunit 2b (Grin2b), and (**F**) the metabotropic glutamate receptor subtype 5 (Grm5). Histograms represent mean ± SEM (n = 10–12 samples per group, half males and half females). Two-way ANOVA and Tukey’s test for multiple comparisons were performed: (*) *p* < 0.05, (**) *p* < 0.01, (***) *p* < 0.001 versus WT-VEH group; (#) *p* < 0.05, (###) *p* < 0.001, versus 3×Tg-AD-VEH group.

3×Tg-AD mice exhibited a marked increase of the mRNA expression of AMPA receptor subunit Gria1 (Figure 7A) and NMDA subunit 2a (Grin2a, Figure 7D) and a decrease in NMDA subunit 1 (Grin1, Figure 7C), NMDA subunit 2b (Grin2b, Figure 7E) and metabotropic type 5 (Grm5, Figure 7F). DCI treatment resulted in a decrease of the mRNA expression of Gria 1, Gria2 and Grin2a, normalizing its expression with respect to wild-type (Figure 7A,B,D).

### 3.8. Effects of DCI on the Protein Expression of Glutamate Receptors in the Hippocampus of 3×Tg-AD Mice

**Figure 8:**
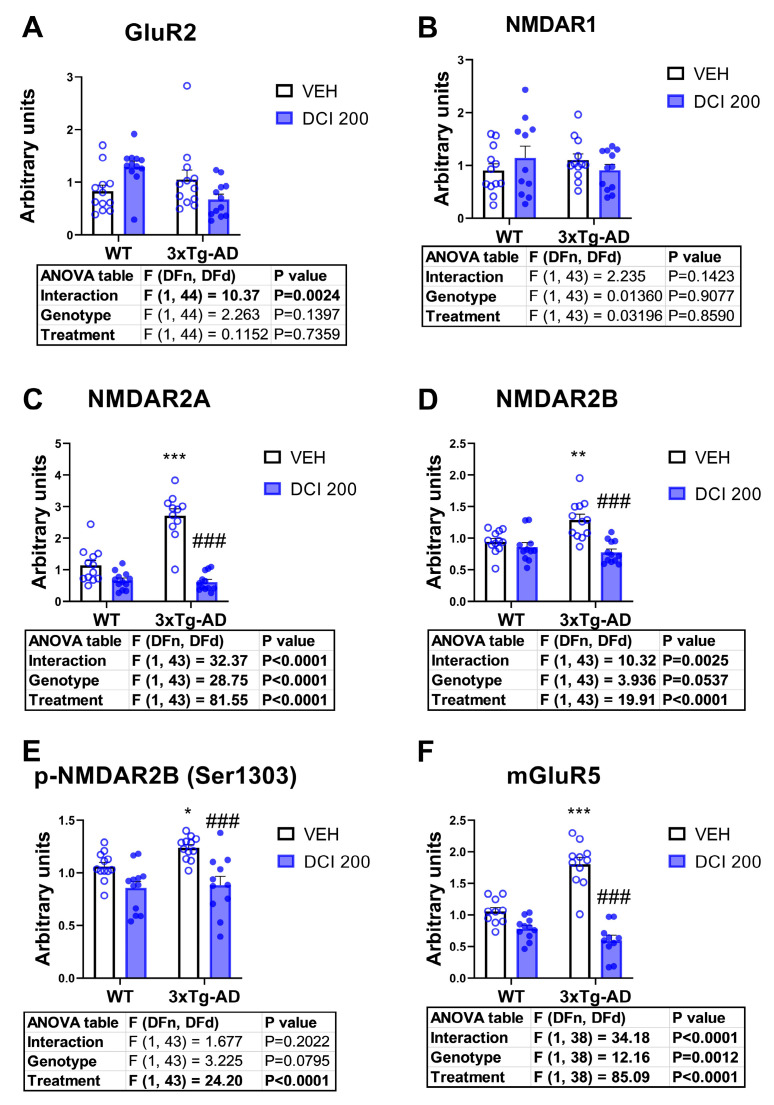
Protein expression of glutamate receptors in the hippocampus of either, wild-type or 3×Tg-AD mice treated for 3 months with D-chiro-inositol (DCI). Western blot analysis measures of (**A**) the glutamate ionotropic receptor AMPA Type Subunit (GluR2), (**B**) the glutamate ionotropic receptor NMDA type subunit 1 (NMDAR1) protein, (**C**) the glutamate ionotropic receptor NMDA type subunit 2a (NMDAR2A), (**D**) the glutamate ionotropic receptor NMDA type subunit 2b (NMDAR2B), (**E**) the serine 1303-phosphorylated form of the glutamate ionotropic receptor NMDA type subunit 2b (*p*-NMDAR2B), and finally, (**F**) the metabotropic glutamate receptor subtype 5 (mGluR5). Histograms represent mean ± SEM (n = 10–12 samples per group, half males and half females). Two-way ANOVA and Tukey’s test for multiple comparisons were performed: (*) *p* < 0.05, (**) *p* < 0.01, (***) *p* < 0.001 versus WT-VEH group; (###) *p* < 0.001, versus 3×Tg-AD-VEH group.

At the protein level, 3×Tg-AD mice exhibited a significant increase in the expression of glutamate receptor subunit NMDA2A (Figure 8C), subunit NMDA2B (Figure 8D), its phosphorylated form at serine 1303 (Figure 8E) and the glutamate metabotropic subtype 5 (Figure 8F). AMPA type receptor subunit 1 (Figure 8A), and glutamate receptor subunit NMDA1 (Figure 8B), did not exhibit genotype-dependent alterations. We were not able to obtain a reliable measure of the GluR1 AMPA receptor subunit. Treatment with DCI reversed the alterations in the expression of glutamate receptor subunit NMDA2A (Figure 8C), subunit NMDA2B (Figure 8D), its phosphorylated form at serine 1303 (Figure 8E) and the glutamate metabotropic subtype 5 (Figure 8F), indicating a DCI-induced normalization of glutamatergic receptor proteins in 3×Tg-AD mice.

### 3.9. Effects of DCI on the mRNA Expression of the Endocannabinoid System in the Hippocampus of 3×Tg-AD Mice

**Figure 9:**
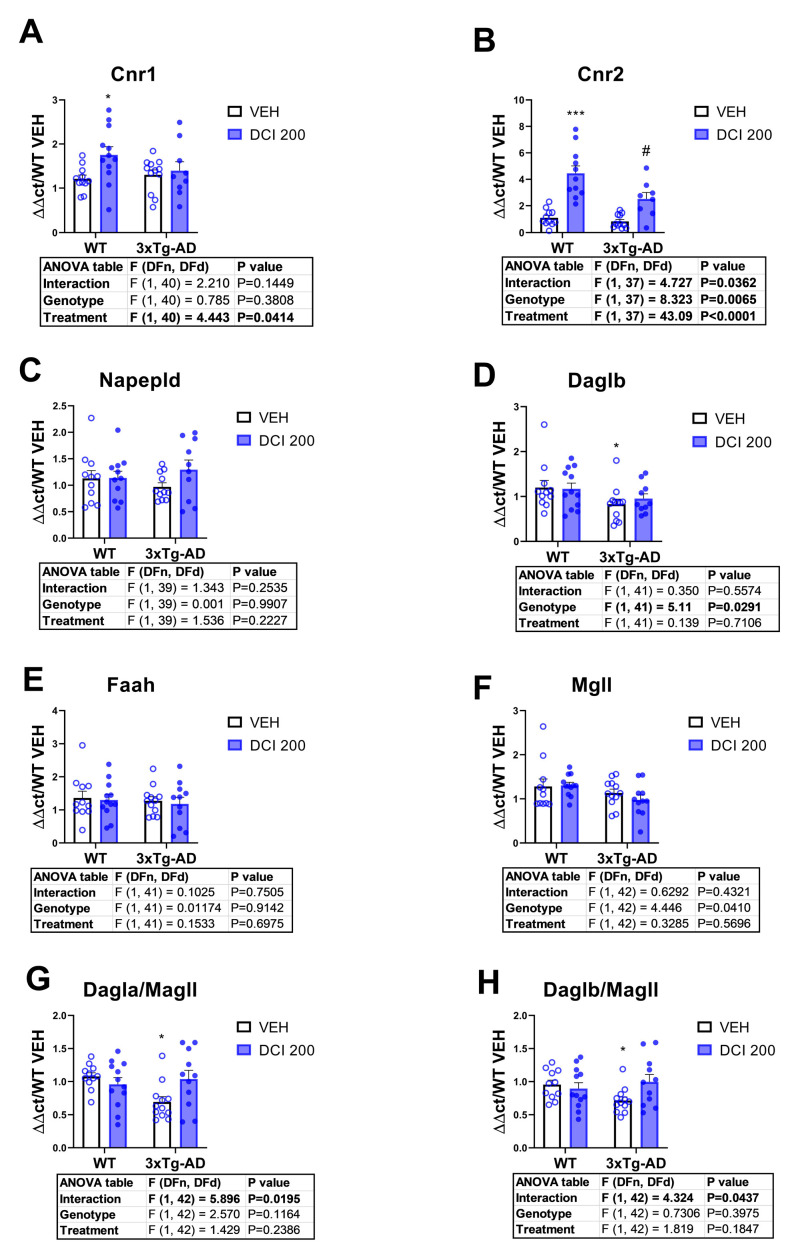
mRNA expression of genes related to endocannabinoid signaling pathway in the hippocampus of either, wild-type or 3×Tg-AD mice treated for 3 months with D-chiro-inositol (DCI). Real-time measurement of the mRNA expression of (**A**) the cannabinoid CB1 receptor (Cnr1), (**B**) the cannabinoid CB2 receptor, (**C**) the enzyme for the synthesis of acylethanolamides, including the endocannabinoid anandamide (Napepld), (**D**) the enzyme for the synthesis of the endocannabinoid 2-AG (Daglb), (**E**) the enzyme degrading acylethanolamides (Faah), (**F**) the enzyme degrading 2-AG, monoacylglycerol lipase (Mgll). The ratio synthesis/degradation of 2-AG, considering (**G**) the isoform a of Dagl and (**H**) the isoform b of Dagl. Histograms represent mean ± SEM (n = 10–12 samples per group, half males and half females). Two-way ANOVA and Tukey’s test for multiple comparisons were performed: (*/***) *p* < 0.05/0.001, versus the WT-VEH group; (#) *p* < 0.05, versus 3×Tg-AD-VEH group.

In order to evaluate the major negative regulatory feedback of glutamatergic transmission, we examined the mRNA expression of proteins involved in endocannabinoid signaling. 3×Tg-AD mice exhibited a clear phenotypic reduction in the endocannabinoid 2-AG production machinery, as revealed by the decrease in the enzyme for its production Daglb (Figure 9D), and the reduction in the ratio production/degradation, measured as the ratio Dagla/Mgll (Figure 9G), and Daglb/Mgll (Figure 9H). Treatment with DCI reversed this decrease in 2-AG degradation machinery (Figure 9D,G,H), and markedly increased CB2 expression in both genotypes (Figure 9B) and CB1 expression in wild-type animals (Figure 9A). The acylethanolamide pathway that generates the endocannabinoid anandamide was not affected by the genotype nor by DCI treatment (Figure 9C,E).

### 3.10. Sex-Dependent Effects of DCI on the Hippocampus of 3×Tg-AD Mice

Although the study of sex differences was not the aim of the present study, some of the effects described above exhibited differential responses regarding sex (See sex analysis at Supplementary Figures S6–S10). Females have a greater response than males to DCI on the expression of insulin-related genes (Figure S6), counteracting the downregulation observed on 3×Tg-AD expression described in Figure 4. Females also have a more significant decrease in GSK-3β expression than males in response to DCI (Figure S7). Regarding the expression of the mRNA of glutamatergic receptors, females exhibited a greater response to DCI, decreasing the enhanced expression of this glutamatergic subunit in the 3×Tg-AD mice (Figure S8). With respect to protein expression, DCI induced a decrease in the protein expression of mGluR5 protein in both genotypes, an effect not observed in males (Figure S9). In the case of the endocannabinoid system, 3×Tg-AD females also exhibited a greater response to DCI, reducing the enhanced levels of expression of Cnr1, Cnr2 and Daglα (Figure S10).

## 4. Discussion

The use of humanized models of familiar Alzheimer’s disease has facilitated research on this chronic neurodegenerative disorder, basically by reducing the time needed for the appearance of the neuropathology and the associated cognitive deficits. However, these models never exhibit a complete overlap with human disorders. Thus, each model helps to select a reduced set of molecular/pathological alterations, facilitating the development of new therapies, but without covering all the aspects of this complex disease. In the present proteomic and phosphoproteomic characterization of the dorsal hippocampus in 12-month-old 3×Tg-AD mice, we found profound disruptions in synaptic organization, mitochondrial bioenergetics, and glutamatergic signaling, all of which converge on mechanisms essential for cognitive function. The dysregulation of mitochondrial oxidative phosphorylation (OXPHOS) components, including Cyc1, Cycs, Sdhb, Cox5a, Uqcrq, Ndufa7, and Atp5d, indicates a multi-complex failure spanning complexes I–V and cytochrome c shuttling. Such defects might compromise ATP synthesis, elevate reactive oxygen species (ROS) production, and disrupt calcium buffering, leading to oxidative stress and increased vulnerability to intrinsic apoptosis [33,34]. In hippocampal neurons—highly reliant on aerobic metabolism—this energy crisis undermines synaptic vesicle cycling, ion homeostasis, and long-term potentiation (LTP), processes central to memory encoding [35]. These mitochondrial deficits are paralleled by a striking reorganization of the glutamatergic synaptic proteome. The altered abundance of vesicle trafficking proteins (Snx1, Rab2a, Itsn2, Arl8a), actin cytoskeletal regulators (Actb, Pak3), and presynaptic scaffolding molecules (Syn2, Erc1) suggests impaired synaptic vesicle docking, release, and recycling, together with destabilization of dendritic spines and postsynaptic receptor clusters [36]. Dysregulation of translational regulators (Eef1a2, Rpl6) and signaling proteins (Pde4a, Fmr1) further points to altered activity-dependent protein synthesis at the synapse, a molecular requirement for enduring synaptic plasticity [37]. Such combined alterations in vesicular trafficking, cytoskeletal dynamics, and local translation are consistent with the synaptic failure considered an early and central event in AD pathogenesis [38]. A third layer of dysfunction emerges from the disruption of proteins governing AMPA-type glutamate receptor (AMPAR) trafficking (Dlg4, Epb41l1, Camk4, Prkcb) and presynaptic release machinery (STX1a, Rims1, Rims2, Snap91). AMPAR endo/exocytosis and membrane stabilization are critical determinants of LTP magnitude and memory persistence [39]. The concurrent dysregulation of cytoskeletal scaffolds (Ank2, Plec, Gphn, Add1, Mical3, Atp1a1) may compromise receptor anchoring and postsynaptic density organization, thereby weakening excitatory drive and synaptic reliability. Given the role of the dorsal hippocampus in spatial learning and declarative memory, these converging proteomic alterations are expected to impair network synchrony, degrade signal fidelity, and diminish the temporal precision of neuronal firing. At the organism level, this would manifest as progressive cognitive decline, reduced adaptability to metabolic or excitotoxic stress, and an increased risk for age-related neurodegenerative progression [8]. Furthermore, the impact may be more pronounced in females, given the known loss of estrogen-mediated mitochondrial and synaptic protection during aging, which is particularly relevant to the epidemiology of AD [34,38].

Together, these findings highlight an integrated failure of mitochondrial metabolism, glutamatergic signaling, and structural synaptic maintenance in the 3×Tg-AD model, underscoring the necessity of therapeutic approaches capable of targeting bioenergetics, oxidative stress, and synaptic plasticity simultaneously. An interesting possibility was to test the efficacy of D-chiro-inositol (DCI), a proposed insulin sensitizer with potential benefits in neurodegenerative disorders [21,24]. To test this possibility, we selected young (3 months old) 3×Tg-AD animals at the beginning of the expression of molecular/neuropathological changes to test the effect DCI, which might help to prevent both insulin deficits and glutamatergic dysfunctions observed in old 3×Tg-AD mice. The present results indicate that the dorsal hippocampus of 3×Tg-AD mice exhibits a pronounced transcriptional downregulation of the canonical insulin signaling pathway, including decreased expression of insulin receptor (IR), IGF-1 receptor (IGF1R), IRS-1, PI3K, AKT2, and GSK-3β mRNAs. Such alterations are consistent with the hippocampal insulin resistance previously described in Alzheimer’s disease (AD) models, where deficits in receptor and kinase expression impair downstream metabolic and synaptic functions [40,41]. Chronic impairment of this pathway reduces PI3K/AKT activation, compromising glucose uptake, mitochondrial function, and neuronal survival [42]. Dietary DCI treatment largely reversed these transcriptional deficits, restoring receptor and substrate expression, and upregulating PI3K specifically in 3×Tg-AD mice. Moreover, DCI increased AKT2 mRNA in both genotypes, suggesting a broader transcriptional effect on metabolic kinases.

At the protein expression level, the hippocampal insulin-signaling phenotype in 3×Tg mice shows a proximal defect at IRS-1—elevated IRS-1 with a trend toward increased inhibitory Ser307 phosphorylation—consistent with impaired receptor–substrate coupling and weakened downstream signal propagation. In this context, D-chiro-inositol (DCI), an insulin sensitizer, reverses several nodes of the deficit: it normalizes the IRS-1/p-IRS-1 balance, increases PI3K abundance, and restores AKT activation (increased p-AKT Ser473) toward physiological levels. These coordinated changes across the IRS-1→PI3K→AKT axis argue for improved pathway competence, i.e., a greater ability of hippocampal neurons to transduce insulin binding into intracellular signaling. The behavior of GSK-3β integrates with, but is not dictated solely by, this proximal rescue. Untreated 3×Tg mice exhibit high basal inhibitory phosphorylation of GSK-3β at Ser9—best interpreted as a maladaptive compensatory “brake” imposed to buffer upstream inefficiency. After DCI, pSer9-GSK-3β shifts toward physiological levels while proximal PI3K/AKT signaling strengthens, suggesting restoration of regulatory flexibility rather than a unidirectional increase or decrease in kinase activity. Importantly, the apparent discrepancy between increased AKT activation and reduced inhibitory GSK-3β Ser9 phosphorylation is mechanistically plausible because Ser9 regulation is not exclusively controlled by the IRS-1/PI3K/AKT pathway. High basal pSer9 in 3×Tg mice can be maintained by kinases other than AKT2 (e.g., PKC, PKA, ERK) or by reduced Ser9 phosphatase activity (e.g., PP1). With DCI, we observe increased activating phosphorylation of AKT2 in both WT and 3×Tg mice and a parallel decrease in GSK-3β Ser9 phosphorylation; together, these data are consistent with DCI shifting the kinase/phosphatase balance, potentially by stimulating a Ser9-directed phosphatase such as PP1 in the hippocampus. Such a shift could dephosphorylate not only GSK-3β but also additional substrates—including tau—thereby aligning with the broader transcriptional and post-translational improvements we observe. Overall, the combined normalization at IRS-1, reinforcement of PI3K/AKT activation, and resetting of GSK-3β Ser9 to physiological, stimulus-responsive control collectively implicate better functioning of hippocampal insulin signaling under DCI treatment in 3×Tg mice. While these findings support a model in which DCI counteracts hippocampal insulin resistance by restoring both upstream signaling capacity and downstream gain control, targeted experiments—e.g., acute insulin challenges ex vivo to probe dynamic responsiveness; assays of PP1 activity and contributions of PKC/PKA/ERK; and linkage to synaptic/tau endpoints—will be valuable to confirm that DCI’s benefits arise from a coordinated rebalancing of kinase–phosphatase networks rather than a simple linear effect within the IRS-1/PI3K/AKT cascade [43,44].

Together, these findings suggest that DCI targets both transcriptional and post-translational levels of insulin signaling regulation, counteracting hippocampal insulin resistance and potentially supporting synaptic integrity and neuroprotection in AD. Interestingly, preliminary sex analysis revealed a potentially better response in female 3×Tg-AD mice, opening the possibility of developing female-oriented interventions in humans.

Concerning glutamatergic dysfunction, a hallmark of Alzheimer’s disease (AD) pathophysiology, contributing to synaptic loss, excitotoxicity, and cognitive decline [45,46], 3×Tg-AD mice displayed significant alterations in the expression of glutamate receptor subunits at both the mRNA and protein levels in the dorsal hippocampus, consistent with previous reports in transgenic AD models [47,48]. At the transcriptional level, 3×Tg-AD exhibited marked upregulation of AMPA receptor subunit Gria1 and NMDA receptor subunit Grin2a, coupled with a reduction in Grin1, Grin2b, and Grm5 mRNA expression. The overexpression of Gria1 and Grin2a may reflect compensatory synaptic responses to early synaptic dysfunction and loss of excitatory inputs [49], whereas the downregulation of Grin1 and Grin2b has been linked to altered NMDA receptor composition, favoring subunit combinations associated with impaired synaptic plasticity and neuroprotection [50]. Reduced Grm5 expression is consistent with evidence that metabotropic glutamate receptor type 5 (mGluR5) participates in amyloid-β–mediated synaptotoxic signaling [51].

At the protein level, we observed increased GluN2A (NMDA2A), GluN2B (NMDA2B), pGluN2B-S1303, and mGluR5, with no significant genotype effect on GluN1 or AMPA GluR1. The elevated phosphorylated GluN2B at Ser1303 is noteworthy, as this modification enhances channel conductance and calcium influx, thereby exacerbating excitotoxic signaling in AD [52]. Interestingly, the divergence between mRNA and protein profiles, particularly for mGluR5 and GluN2B, suggests post-transcriptional regulation, altered receptor trafficking, or impaired proteostasis, all of which have been implicated in AD pathogenesis [53].

DCI treatment for three months normalized several of these alterations. At the mRNA level, DCI reduced Gria1, Gria2, and Grin2a expression, restoring values to wild-type levels. At the protein level, DCI attenuated the elevated GluN2A, GluN2B, pGluN2B-S1303, and mGluR5, suggesting a broad modulatory effect on glutamatergic receptor homeostasis. The normalization of pGluN2B-S1303 is particularly significant, as excessive phosphorylation of this site has been associated with synaptic loss and cognitive impairment [54]. Mechanistically, DCI is an inositol stereoisomer involved in phosphoinositide signaling, insulin sensitization, and calcium homeostasis [21,22,23,24]. Its capacity to modulate glutamate receptor expression could be mediated through the PI3K–AKT–mTOR pathway, which influences receptor trafficking and synaptic plasticity [55]. By restoring glutamatergic receptor balance, DCI may mitigate excitotoxicity while preserving physiological NMDA and AMPA receptor signaling, thus supporting synaptic resilience in the hippocampus. Taken together, these findings suggest that DCI exerts neuroprotective effects in the 3×Tg-AD hippocampus by normalizing pathological glutamate receptor expression at both transcriptional and translational levels. Given the dual role of glutamate receptors in learning and excitotoxic damage, the modulatory profile of DCI positions it as a potential therapeutic agent for correcting synaptic signaling imbalances in AD. Future studies should address whether these molecular changes translate into functional improvements in synaptic transmission and memory performance.

To further explore the modulation of dysfunctional glutamatergic synapses by DCI in the 3×Tg-AD mice, we explored the expression of the mRNA of the endocannabinoid system (eCB) in the hippocampus. The eCB system is a pivotal retrograde signaling mechanism that modulates hippocampal glutamatergic transmission by inhibiting presynaptic neurotransmitter release via CB1 receptor activation [56,57]. This negative feedback loop is essential for maintaining excitatory–inhibitory balance and preventing glutamate-induced excitotoxicity [58]. In the present study, 3×Tg-AD mice exhibited a pronounced downregulation of the 2-arachidonoylglycerol (2-AG) production machinery, specifically diacylglycerol lipase β (Daglb), along with reduced Dagla/Mgll and Daglb/Mgll ratios, indicating impaired capacity for 2-AG synthesis relative to its degradation. Such alterations suggest a compromised retrograde inhibitory control of glutamate release, which could exacerbate the glutamatergic overdrive already documented in these mice at both the mRNA and protein levels for receptor subunits.

Importantly, DCI treatment reversed the reduction in Daglb expression and restored the production/degradation ratios toward wild-type levels, suggesting a normalization of 2-AG–mediated presynaptic regulation. This restoration is particularly relevant given that deficient 2-AG signaling has been shown to worsen glutamate-driven neurotoxicity and accelerate synaptic deterioration in AD models [59,60]. Furthermore, DCI markedly increased CB2 receptor mRNA expression in both genotypes and elevated CB1 receptor mRNA expression in wild-type mice. While CB1 is predominantly expressed at glutamatergic presynaptic terminals in the hippocampus and directly mediates suppression of excitatory transmission [61], CB2 is mainly found in glial cells and is associated with anti-inflammatory and neuroprotective actions [62]. The upregulation of CB2 could therefore indicate a DCI-induced shift toward a more anti-inflammatory glial phenotype, potentially reducing neuroinflammation-driven synaptic dysfunction [63]. The CB1 increase in wild-type mice—but not in 3×Tg-AD animals—may suggest that chronic pathology in the latter blunts receptor plasticity, even when upstream 2-AG synthesis is partially restored.

Notably, the anandamide (AEA) pathway remained unaffected by genotype or treatment, indicating that the observed DCI effects on eCB signaling are primarily mediated through the 2-AG pathway. Given that 2-AG, rather than AEA, is the main retrograde modulator of hippocampal glutamate release [56,64], these findings reinforce the functional relevance of DCI’s modulation of DAGL–MAGL balance. When considered alongside the glutamatergic receptor data, the present results suggest that DCI’s neuroprotective profile in the 3×Tg-AD hippocampus arises from a dual action: a) Normalizing postsynaptic receptor expression (AMPA, NMDA, mGluR5), thereby preventing receptor-mediated excitotoxicity and b) Restoring presynaptic inhibitory tone through enhanced 2-AG production capacity, improving retrograde suppression of glutamate release.

This combined action could effectively reduce aberrant excitatory drive, a critical pathogenic mechanism in AD, and promote synaptic stability. Future electrophysiological studies should assess whether these molecular effects translate into restored short- and long-term synaptic plasticity, such as normalization of depolarization-induced suppression of excitation (DSE) [65], which is known to be impaired in AD.

A key limitation of this study is the reliance on the 3×Tg-AD mouse model, which, although useful for examining accelerated amyloidosis and tau deposition, does not recapitulate the full spectrum of Alzheimer’s disease pathogenesis in humans, particularly in sporadic cases. A second limitation concerns the timing: outcomes were assessed at ~6 months of age (end of DCI treatment), a stage at which tauopathy is not yet fully developed. Finally, our analyses focused on the molecular layer and did not include functional assessments linking restoration of molecular markers to normalization of function (e.g., electrophysiological or behavioral readouts). Future studies should address the functional recovery of the phenotype.

## 5. Conclusions

Despite this limitation, the pharmacological profile of DCI supports its potential use in mitigating various factors that contribute to cognitive impairment, particularly in the early stages of the disease. Furthermore, these results position DCI as a potential disease modifier and highlight its utility as a tool for the development of new AD treatments. DCI could be combined with other therapeutic strategies, such as immunotherapies targeting Aβ, to enhance treatment outcomes.

## Data Availability

A detailed protocol outlining the research question, key design features, and analysis plan was prepared before the commencement of the study. This protocol was not registered in any public repository. This published article and its Supplementary Information files include all data generated or analyzed during this study. Raw data are available upon reasonable request from the corresponding author.

## Supplementary Materials

The following supporting information can be downloaded at: https://www.mdpi.com/article/10.3390/nu17183024/s1, Table S1: Primer references for qPCR. Table S2: Primary antibodies used for protein expression by Western blotting; Figure S1: Gene Ontology analysis of deregulated proteins in the hippocampus; Figure S2: Gene Ontology analysis of deregulated phosphoproteins in the hippocampus. Figures S3–S5: Whole-membrane gels (unedited blots) of Western blot analysis; Figure S6: Sex differences in the mRNA expression of genes related with insulin signaling in the hippocampus. Figure S7: Sex differences in the phosphorylation and total expression of proteins related to insulin signaling in the hippocampus; Figure S8: Sex differences in the mRNA expression of genes related to glutamate signaling in the hippocampus. Figure S9: Sex differences in the total expression of proteins related to glutamate signaling in the hippocampus. Figure S10: Sex differences in the mRNA expresion of genes related with endocannabinoid signaling in the hippocampus.
