# Supplementary material for: Early Oral Administration of D-Chiro-Inositol Reverses Hippocampal Insulin and Glutamate Signaling Deficits in the 3×Tg Humanized Mouse Model of Alzheimer’s Disease
Source: Nutrients. 2025 Sep 22;17(18):3024. doi: 10.3390/nu17183024 (PMC12472782; doi:10.3390/nu17183024)

PACHECO-SANCHEZ, B ET AL.
SUPPLEMENTARY MATERIALS

1. SUPPLEMENTARY TABLES
2. SUPPLEMENTARY FIGURES

Supplementary Table S1. Primer references for TaqMan® Gene Expression Assays (Thermo Fisher Scientific).

Gene ID	GenBank accession nº	Assay ID	Amplicon length
Actb	NM_007393.5	Mm02619580_g1	143
Aif1	NM_019467.2	Mm00479862_g1	82
Akt2	NM_001110208.2	Mm05804786_gH	75
Cnr1	NM_007726.3	Mm01212171_s1	66
Cnr2	AK134109.1	Mm02620087_s1	171
Daglb	NM_144915.3	Mm00523381_m1	72
Faah	NM_010173.4	Mm00515684_m1	62
Gfap	NM_001131020.1	Mm01253033_m1	75
Gpr55	NM_001033290.2	Mm02621622	102
Gria1	NM_001113325.2	Mm00433752_m1	66
Gria2	NM_001039195.3	Mm00442822_m1	67
Grin1	NM_001177656.2	Mm00433790_m1	63
Grin2a	NM_008170.4	Mm00433802_m1	84
Grin2b	NM_008171.4	Mm00433820_m1	61
Grm5	NM_001081414.2	Mm00690332_m1	95
Gsk3ß	NM_019827.7	Mm00444911_m1	72
Igf1r	NM_010513.2	Mm00802831_m1	106
Insr	NM_010568.2	Mm01211879_m1	102
Irs1	NM_010570.4	Mm01278327_m1	69
Mgll	NM_001166249.1	Mm04206876_m1	102
Napepld	NM_178728.5	Mm00724596_m1	85
Pik3r	NM_001024955.2	Mm01282781_m	74
Ppara	NM_001113418.1	Mm00440939_m1	74

Supplementary Table S2. Primary antibodies used for protein expression by western blotting

ANTIGEN	MANUFACTURING DETAILS	DILUTION
p-Irs1 (Ser307)	Cell Signaling Technology (#2381)	1:1000
Irs1	Cell Signaling Technology (#3407)	1:1000
p-PI3K (Y607)	Abcam (#ab182651)	1:500
PI3K	Cell Signaling Technology (#4257)	1:1000
p-Akt (Ser473)	Cell Signaling Technology (#9271)	1:1000
Akt	Cell Signaling Technology (#9272)	1:1000
p-GSK-3β (Ser9)	Cell Signaling Technology (#5558)	1:1000
GSK-3β	Cell Signaling Technology (#12456)	1:1000
GluR2	Abcam (#ab 206293)	1:500
NMDAR1	Abcam (#ab109182)	1:500
NMDAR2A	Abcam (#ab183939)	1:500
p-NMDAR2B (S1003)	Abcam (#ab81271)	1:500
NMDAR2B	Sigma-Aldrich (# 06-600)	1:500
mGluR5	Abcam (#ab76316)	1:500
IBA1	Abcam (#ab5076)	1:1000
GFAP	Sigma-Aldrich (#G3893)	1:500
γ-Adaptin	bd biosciences (#610385)	1:2000

Abbreviations: Insulin receptor substrate 1: Irs1, Phosphatidylinositol 3-kinase: PI3K, Protein kinase B: Akt, Glycogen synthase kinase-3 beta: GSK-3β, Glutamate ionotropic receptor AMPA type subunit 2: GluR2, N-methyl-D-aspartate (NMDA) Receptor 1: NMDAR1, N-methyl-D-aspartate (NMDA) Receptor Subunit 2A: NMDAR2A, N-methyl-D-aspartate (NMDA) Receptor Subunit 2B: NMDAR2B, Metabotropic glutamate receptor 5: mGluR5, Ionized calcium-binding adapter molecule 1: IBA1 and Glial fibrillary acidic protein: GFAP.

Pacheco et al.
SUPPLEMENTARY FIGURES

1. **ADDITIONAL PROTEOMICS ANALYSIS**
2. **REPRESENTATIVE GELS**
3. **ANALYSIS OF SEX DIFFERENCES**

Supplementary Figure S1. Gene Ontolology analysis of proteins deregulated in the hippocampus of 3XTG mice

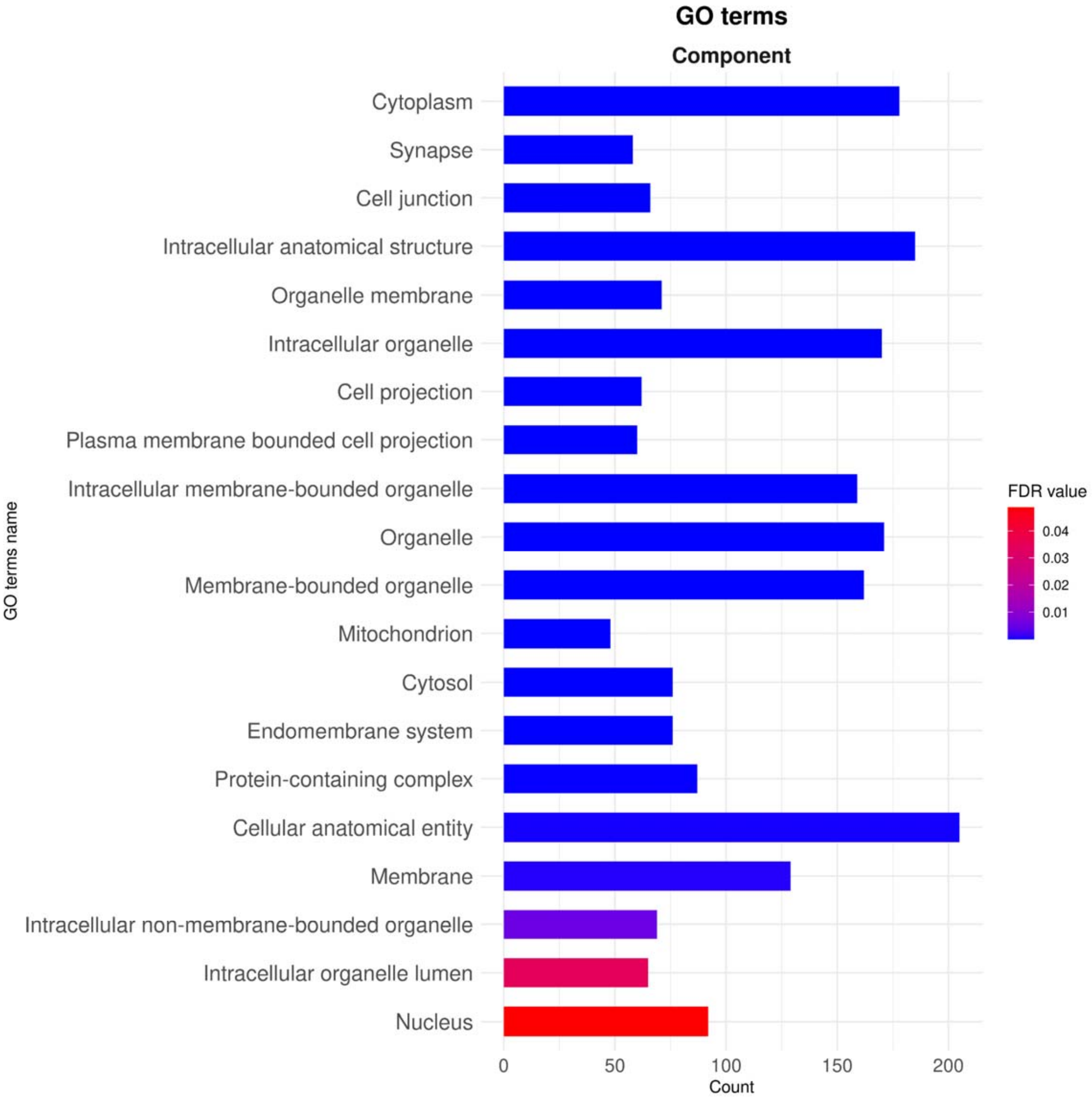

Supplementary Figure S2. Gene Ontolology analysis of phosphoproteins deregulated in the hippocampus of 3XTG mice

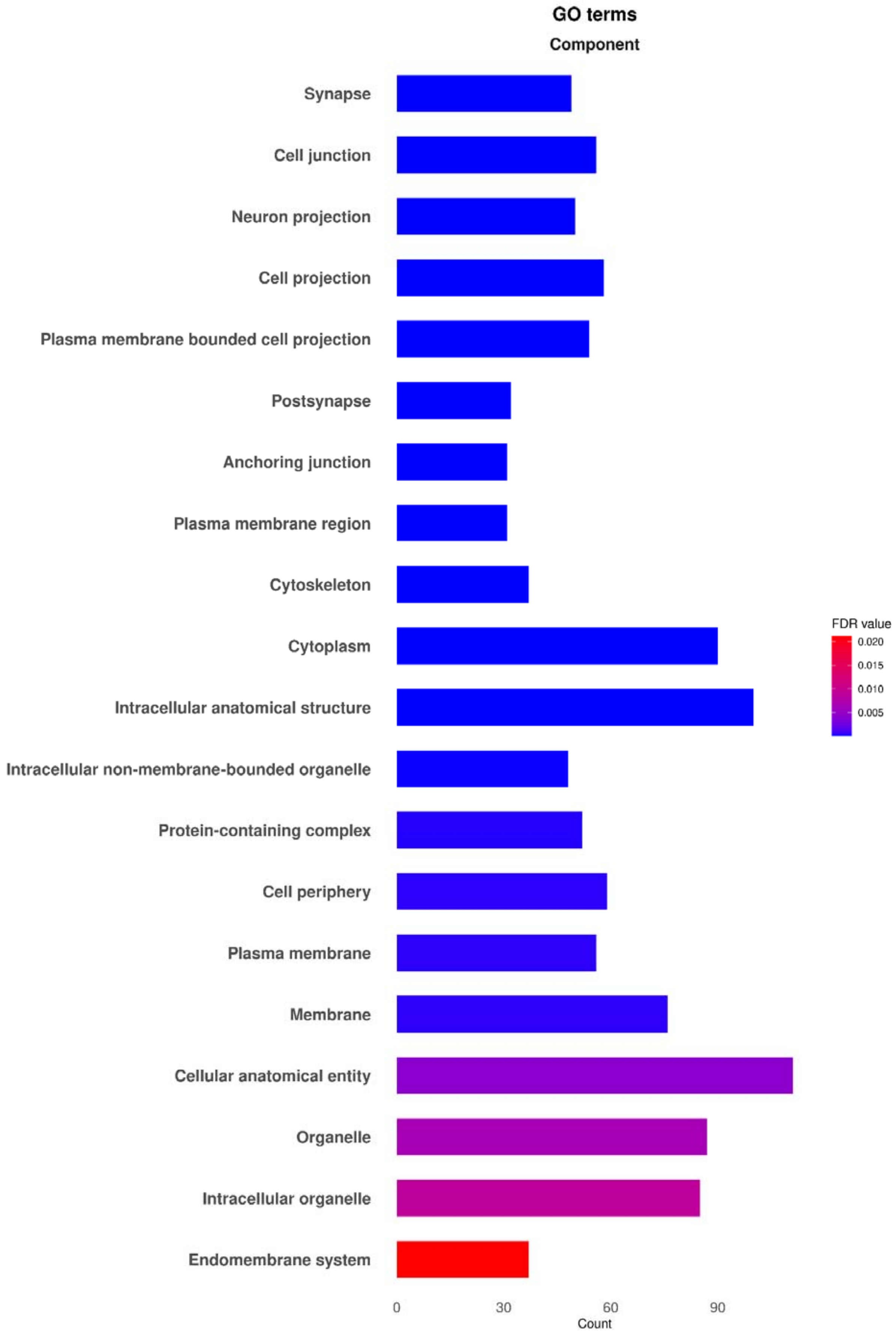

Supplementary Figure S3. Representative Western blot images from Figure 5.

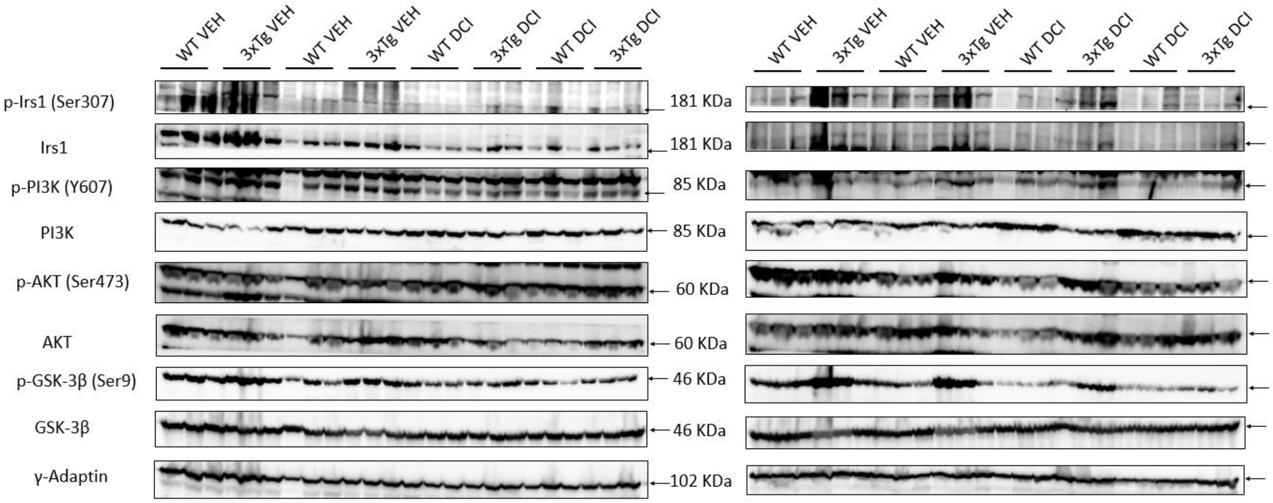

Supplementary Figure S4. Representative Western blot images from Figure 6.

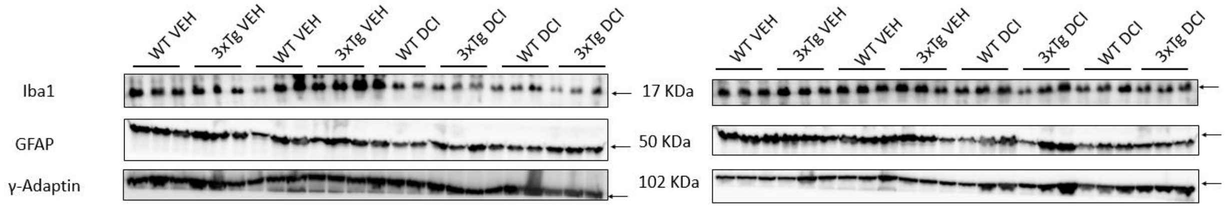

Supplementary Figure S5. Representative Western blot images from Figure 8.

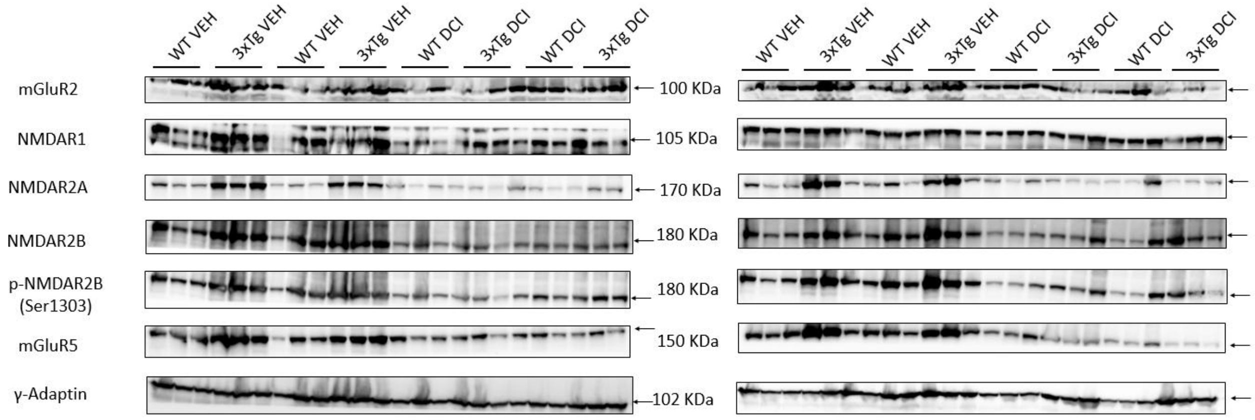

Supplementary Figure S6. Sex differences in the mRNA expression of genes related with insulin signaling in the hippocampus

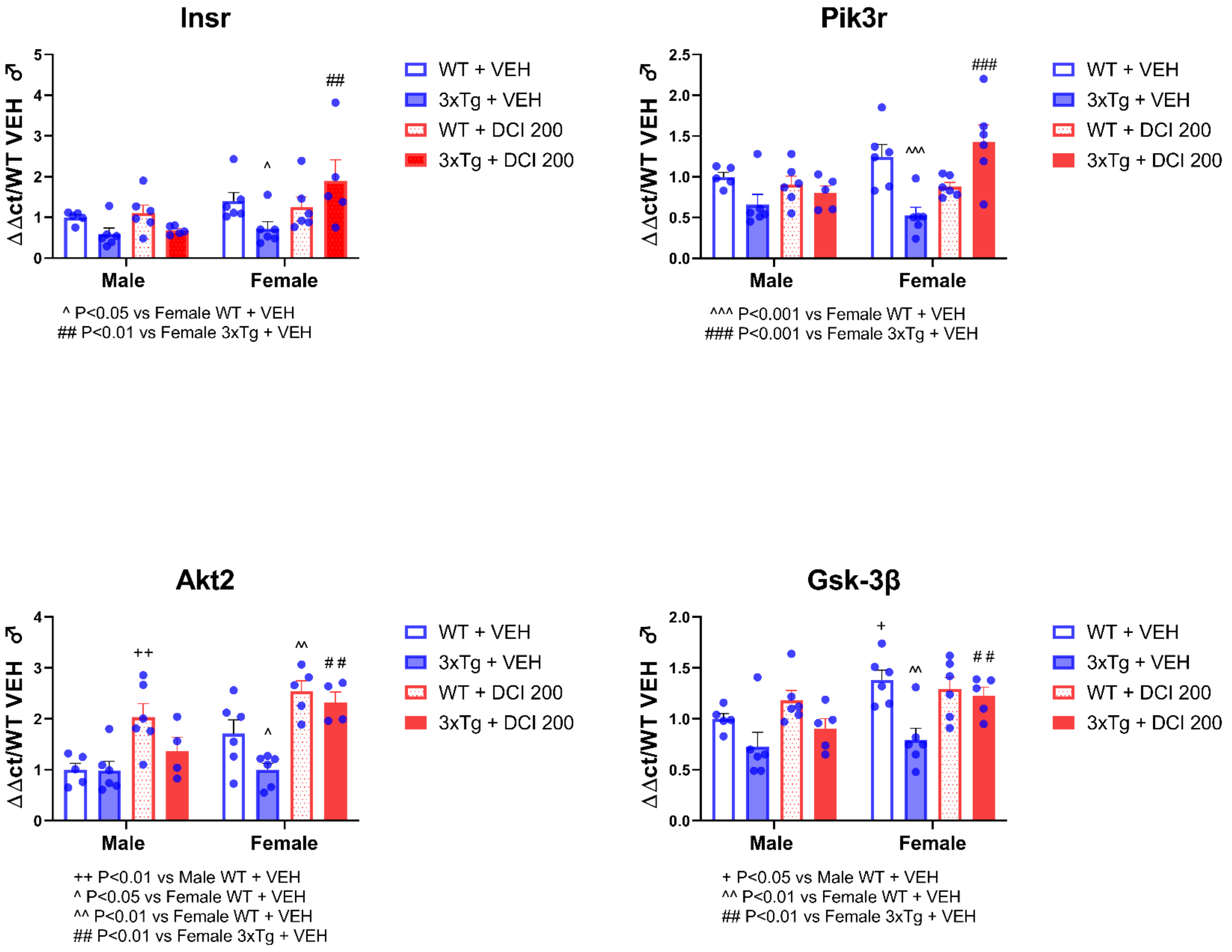

Supplementary Figure S7. Sex differences in the phosphorylation and total expression of proteins related with insulin signaling in the hippocampus

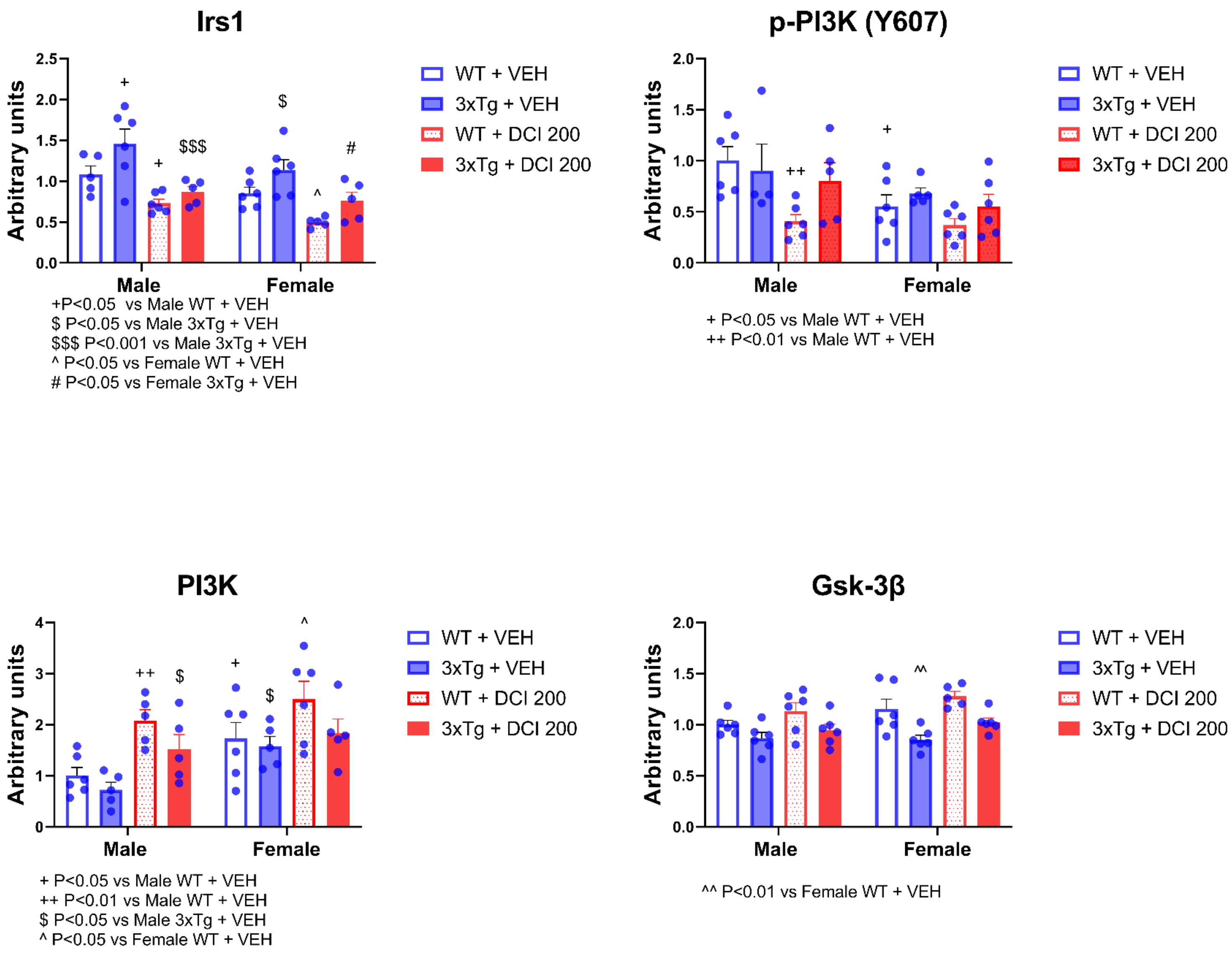

Supplementary Figure S8. Sex differences in the mRNA expression of genes related with glutamate signaling in the hippocampus

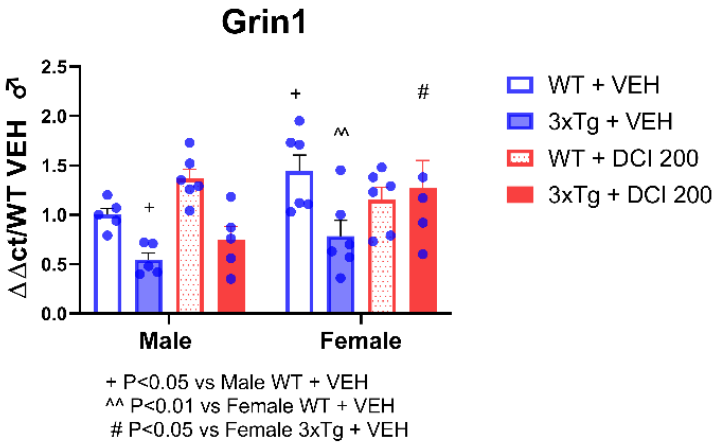

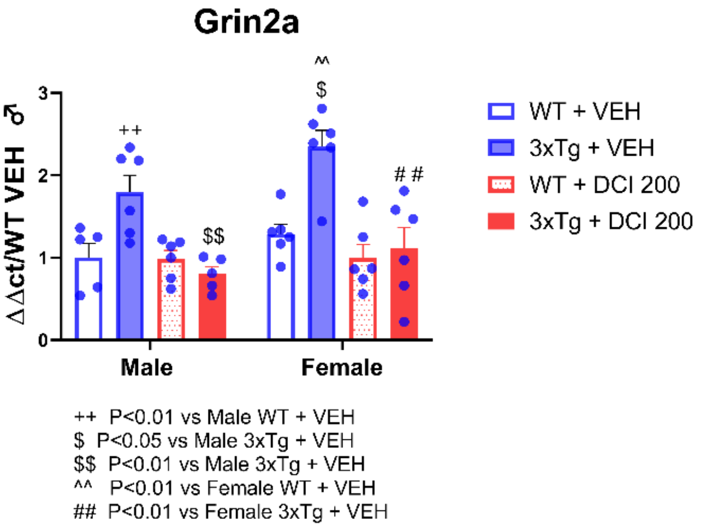

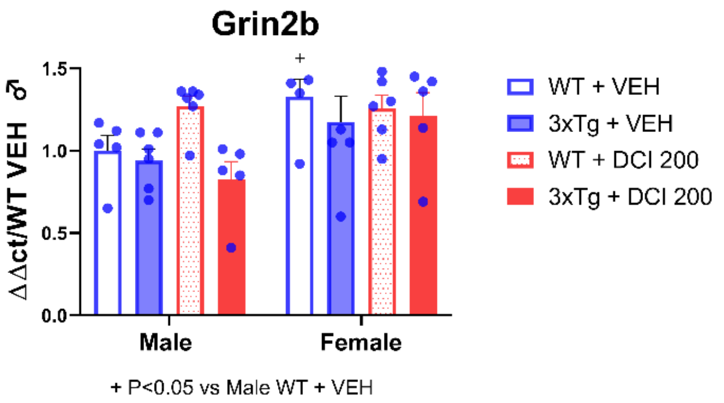

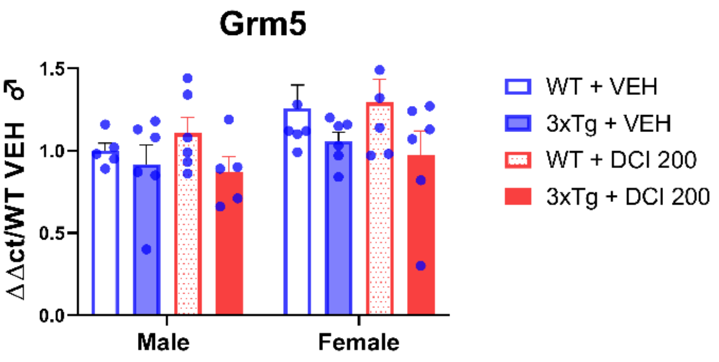

Supplementary Figure S9. Sex differences in the total expression of proteins related with glutamate signaling in the hippocampus

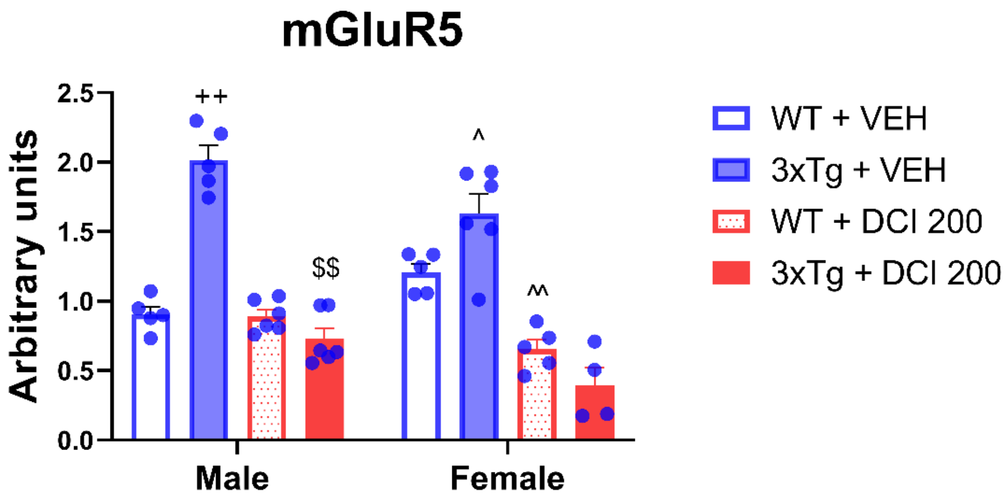

++ P<0.01 vs Male WT + VEH
$$ P<0.01 vs Male 3xTg + VEH
^ P<0.05 vs Female WT + VEH
^^ P<0.01 vs Female WT + VEH

Supplementary Figure S10. Sex differences in the mRNA expression of genes related with endocannabinoid signaling in the hippocampus

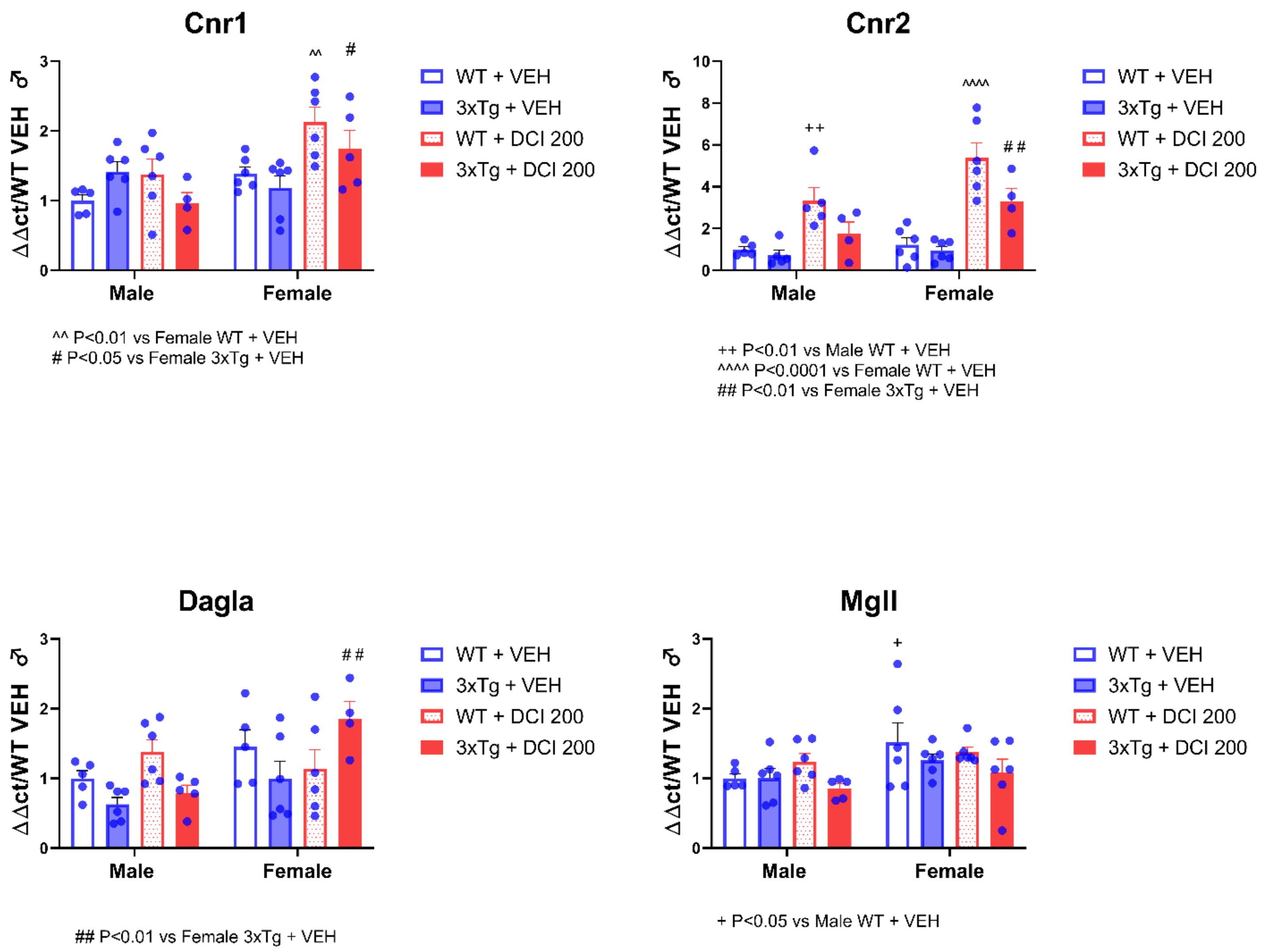

Supplement: Supplementary file 1 [file nutrients-17-03024-s001.zip › nutrients-3845096-supplementary.pdf]
